# Identification of novel biomarkers for anti-*Toxoplasma gondii* IgM detection and the potential application in rapid diagnostic fluorescent tests

**DOI:** 10.3389/fmicb.2024.1385582

**Published:** 2024-06-04

**Authors:** Minh-Ngoc Nguyen, Seon-Ju Yeo, Hyun Park

**Affiliations:** ^1^Department of Infection Biology, School of Medicine, Zoonosis Research Center, Wonkwang University, Iksan, Republic of Korea; ^2^Department of Tropical Medicine and Parasitology, Department of Biomedical Sciences, College of Medicine, Seoul National University, Seoul, Republic of Korea; ^3^Department of Tropical Medicine and Parasitology, Medical Research Center, Institute of Endemic Diseases, Seoul National University, Seoul, Republic of Korea

**Keywords:** *Toxoplasma gondii*, diagnosis, IgM detection, point-of-care test, fluorescence immunochromatographic test, 2DE immunoblotting

## Abstract

Toxoplasmosis, while often asymptomatic and prevalent as a foodborne disease, poses a considerable mortality risk for immunocompromised individuals during pregnancy. Point-of-care serological tests that detect specific IgG and IgM in patient sera are critical for disease management under limited resources. Despite many efforts to replace the *T. gondii* total lysate antigens (TLAs) by recombinant antigens (rAgs) in commercial kits, while IgG detection provides significant specificity and sensitivity, IgM detection remains comparatively low in sensitivity. In this study, we attempted to identify novel antigens targeting IgM in early infection, thereby establishing an IgM on-site detection kit. Using two-dimensional gel electrophoresis (2DE) and mouse serum immunoblotting, three novel antigens, including EF1γ, PGKI, and GAP50, were indicated to target *T. gondii* IgM. However, rAg EF1γ was undetectable by IgM of mice sera in Western blotting verification experiments, and ELISA coated with PGKI did not eliminate cross-reactivity, in contrast to GAP50. Subsequently, the lateral flow reaction employing a strip coated with 0.3 mg/mL purified rAg GAP50 and exhibited remarkable sensitivity compared with the conventional ELISA based on tachyzoite TLA, which successfully identified IgM in mouse sera infected with tachyzoites, ranging from 10^3^ to 10^4^ at 5 dpi and 10^4^ at 7 dpi, respectively. Furthermore, by using standard *T. gondii*-infected human sera from WHO, the limit of detection (LOD) for the rapid fluorescence immunochromatographic test (FICT) using GAP50 was observed at 0.65 IU (international unit). These findings underline the particular immunoreactivity of GAP50, suggesting its potential as a specific biomarker for increasing the sensitivity of the FICT in IgM detection.

## 1 Introduction

*Toxoplasma gondii* is a unicellular spore-forming organism that is an obligate endoparasite in virtually all warm-blooded animals. It is estimated that one-third of people across the world are exposed to *T. gondii*, mainly through consuming raw and contaminated foodstuffs or accidentally swallowing the parasite after coming in contact with cat feces (Dubey, 2010; Lourido, 2019). Toxoplasmosis is typically asymptomatic but is a prevalent foodborne disease that leads to mortality because of the development of severe clinical symptoms in immunocompromised individuals, such as AIDS patients, those undergoing chemotherapy, organ transplantation recipients, or infants born to mothers who were recently infected with *T. gondii* during or just before pregnancy (Ajzenberg et al., 2009; Centers for Disease Control and Prevention, 2023). There is minimal risk if the mother acquired the infection before conception within a few months, but risks increase in later stages, potentially causing preterm delivery or fatalities. Congenital infection is common in the last trimester, with newborns often asymptomatic at birth but susceptible to suffering later symptoms such as blindness or mental disorders (Montoya and Liesenfeld, 2004; Arranz-Solis et al., 2021). Early diagnosis in pregnant women with disease onset reduces congenital transmission in neonates and allows for timely treatment.

Toxoplasmosis is mainly diagnosed using serological tests that detect specific IgG and IgM antibodies in the patient’s sera. Anti-*T. gondii*-specific IgG appears nearly 2 weeks after pathogen contact and persists for a long time, making it useful for assessing whether a person has been infected (Montoya and Liesenfeld, 2004; Teimouri et al., 2020). On the other hand, IgM typically appears earlier than IgG and experiences a dramatic increase in levels that peaks after 2–3 weeks of infection before declining to background at 2–3 months (Turunen et al., 1983; Trees et al., 1989; Teimouri et al., 2020). However, unlike other infectious diseases, it became evident that anti-*T. gondii* IgM responses can persist for months or even years, as reported in numerous clinical cases (Bobic et al., 1991; Vargas-Villavicencio et al., 2022). Although other methods such as IgG avidity may need accurate determination of acute infection, the assessment of specific IgM remains mandatory (Reis et al., 2006; Rahimi-Esboei et al., 2018).

The first-line point-of-care (POC) commercial tests are designated Toxoplasma ICT IgG-IgM (LDBIO Diagnostic, Lyon, France), which integrates the tachyzoites total lysate antigen (TLA) derived from mouse proliferation or *in vitro* tissue cultures, providing 100% sensitivity and specificity in the USA, while sensitivity and specificity were reported to be 97 and 96%, respectively, in France (Begeman et al., 2017; Chapey et al., 2017; Khan and Noordin, 2020). Nevertheless, using TLA in immunoassays is time-consuming, expensive, and difficult to standardize during propagation and lysate preparation (Ybanez et al., 2020).

Thus, many efforts have been made to replace TLA with recombinant antigens (rAgs) for the diagnosis of toxoplasmosis in humans and animals (Holec-Gasior, 2013). These include antigens from the parasite surface (SAGs), matrix (MAGs), dense granules (GRAs), rhoptry (ROPs), micronemes (MICs), and even a combination of several antigens and chimeric antigens (Khan and Noordin, 2020).

Two commercial POC kit: Biopanda Toxo IgG/IgM or OnSite Toxo IgG/IgM tests, both of which use colloidal gold-conjugated recombinant proteins, provide 100% sensitivity and 96.3 and 97.5% specificity, respectively, for IgG detection. However, IgM detection using the Biopanda kit is only 88.5% specific and 62.2% sensitive, while IgM detection using the OnSite kit is only 97.6% specific and 28% sensitive (Gomez et al., 2018; Khan and Noordin, 2020). Sometime natural IgM antibodies produce false positives when reacting with *T. gondii* antigens in the absence of infection (Liesenfeld et al., 1997). Although the LDBIO has great sensitivity, it uses a single line-coated TLA to detect both IgG and IgM antibodies on the strip, whereas the Biopanda and OnSite tests employ separate lines for each, allowing for further assessment of acute or chronic infections (Begeman et al., 2017; Chapey et al., 2017; Gomez et al., 2018). Thus, since rAgs have not yet entirely replaced native tachyzoite antigens in POC testing, improvement in the toxoplasmosis IgM diagnosis using rAg is needed.

Due to clinical silence of *T. gondii*, in most cases, studies on the estimation of disease onset often face limitations of insufficient data (Nayeri et al., 2020). To address this challenge, animal models of infected parasites can imitate human-like immune responses and provide insights into the timeline of disease onset (Flegr et al., 2014; Anand et al., 2015; Poshtehban et al., 2017; Doskaya et al., 2018; Lutshumba et al., 2020; Anand et al., 2022). Cysts of the 76 K strain are produced antibodies after 2 weeks, peaking at 6 weeks post-infection (wpi). The IgG titer remains constant until 8 wpi, while the IgM titer rapidly declines after 7 wpi (Kang et al., 2006). Mice exposed to oocysts or tissue cysts exhibited a robust IgM response by day 10, but their IgM dropped in oocyst-infected mice and rose in tissue cyst-infected mice until day 15, suggesting that bradyzoites from tissue cysts penetrate host cells for a long time (Doskaya et al., 2018). In comparison, both virulent (Ck2) and non-virulent (ME49) strains exhibited increased IgM reactivity during in the first 15, but the decline in IgM levels of virulent strain was faster than in the non-virulent strain (Alvarado-Esquivel et al., 2011; Oliveira et al., 2016). Experimental infection of dogs with the virulent *T. gondii* RH strain (Silva et al., 2002) showed a similar pattern. Despite limited IgM monitoring in animals, often lasting up to 10 wpi with unclear long-term duration due to sporadic trials (Vargas-Villavicencio et al., 2022), rodent models offer a controlled environment for studying infection progression.

In the present study, using two-dimensional gel electrophoresis (2DE), mice sera immunoblotting, and mass spectrometry (MS) analysis, we attempted to discover three previously uncharacterized antigens of *T. gondii*, EF1γ, PGKI, and GAP50—which have the potential to distinctly target *T. gondii* IgM antibodies during the early infection of rodent model. The diagnostic capabilities of the novel generated rAgs were assessed using immunoblot analysis and, ultimately, a rapid fluorescence immunochromatographic test (FICT) to detect IgM in both experimentally infected mice and human serum samples.

## 2 Materials and methods

### 2.1 Parasites and animals

Tachyzoites of a high-virulence RH *T. gondii* strain (ATCC PRA-310) were maintained *in vitro* by infection of the APRE19 cell line (ATCC CRL-2302) and cultured in DMEM/F-12 medium (LM002-04, Welgene, Korea) containing 2% of fetal bovine serum (FBS) (16000-044, Gibco, Thermo Fisher Scientific) and 1% penicillin–streptomycin (15140–122, Gibco Thermo Fisher Scientific). Infected cells were cultured at 37°C in a 5% CO_2_ atmosphere, and the fresh medium was changed every 2–3 days until tachyzoites were harvested.

### 2.2 *Toxoplasma gondii* TLA preparation

The supernatant and scraped infected cells were centrifuged at 1,200*g* for 10 min at room temperature (RT) to collect the parasites, which were then repeatedly passed through a 25 gauge needle. Tachyzoites were subsequently isolated by gradual and careful layering them on a 40% Percoll (GE17089101, MERK) solution and centrifuging at 1,200*g* for 20 min at RT. Following the removal of Percoll, the parasite was washed three times in PBS (ML 008-01, Welgene, Korea) to remove cell debris.

### 2.3 Sera from mice infected with *Toxoplasma gondii*

8–12-week-old female BALB/c mice were purchased from Orient Bio (Seongnam, Gyeonggi, Korea) and were maintained under conventional conditions. Mice from each group (*n* = 5) were intraperitoneally (i.p.) inoculated with 10^1^–10^6^ tachyzoites of RH strains per mouse. After 2–10 days of infection, mouse blood was collected and sera were obtained for further experiments (Greenfield, 2017). Negative controls included sera from uninfected BALB/c mice of the same age and sex (*n* = 10), and additional serum groups of *P. yoelii* (*n* = 10) were used to assess cross-reactivity. *P. yoelii* was i.p. injected in the mouse belly with 10^4^ infected RBCs per mouse, and sera from blood were collected on day 5 post-infection at 20–30% parasitemia.

### 2.4 Human sera

The WHO International Standard 4th IS for Antibodies to *Toxoplasma gondii* in human plasma (NIBSC code 13/132) is a reference reagent comprising 160 IU (international unit)/ 0.5 mL of pooled plasma obtained from six donors who were recently infected with *T. gondii*. This freeze-dried preparation contains a significant amount of both IgG and IgM antibodies and is used as a benchmark for diagnostic testing (Rijpkema et al., 2016; NIBSC, 2020).

Healthy individual samples (toxoplasmosis-negative sera) (*n* = 10), *P. vivax*-infected samples (*n* = 10), and a Dengue Mixed Titer Accuset Performance Panel (0845-0074, SeraCare) (*n* = 10) were used as additional comparable serum samples.

### 2.5 Two-dimensional gel electrophoresis (2DE)

TLA of *T. gondii* tachyzoites and bradyzoites mixture compared with APRE19 mock-infected cells was prepared and dissolved in 500 μL of sample buffer (7 M urea, 2 M thiourea, 4% CHAPS, 65 mM DTT, and 2% IPG buffer, pH 3–10 NL (GE17-6000-88, MERK)) and then incubated on ice for 60 min. Freezing and thawing the sample with liquid N_2_ were repeated three times, followed by a sonication step for 5 min on ice (1 s on, 5 s off, 40 Amp) and centrifugation at 17,000 rpm for 45 min at 4°C to collect the supernatant. Subsequently, the protein supernatant was subjected to the Readyprep 2D cleanup kit (163-2130, Bio-Rad), following the manufacturer’s instructions. The final purified pellet was re-suspended in 200 μL of sample buffer containing 0.04% w/v bromophenol blue and loaded onto either a ReadyStrip IPG Strip 7 cm pH 3–10 NL (163-2002, Bio-Rad) or Readystrip IPG strip, 7 cm, pH 5–8 (163-2004, Bio-Rad) for rehydration at 50 V for 12–16 h at 20°C using the PROTEAN IEF Cell system (165-4000, Bio-Rad).

All bubbles were removed from underneath the IPG strip before adding 1 mL of strip cover fluid mineral oil (163-2129, Bio-Rad). Subsequently, isoelectric focusing was carried out using a multistep protocol on a 7-cm strip: 250 V for 15 min in linear voltage mode, followed by 1,000 V for 30 min, 4,000 V for 2 h, and then 4,000 V for 5 h in rapid voltage mode. An optional step at 50 V for 15 h was also included.

After the focusing step was completed, individual strips were removed from the IEF machine and then equilibrated for 25 min with equilibrated buffer (1.5 M Tris HCl, pH 8.8, 6 M urea, 30% glycerol, 2% SDS, and 0.04% bromophenol blue) containing 65 mM DTT followed by additional equilibration for 25 min with the same buffer as used for DTT with 135 mM iodoacetamide. For the second dimension, proteins were separated by SDS-PAGE in a 10% resolving gel. Electrophoresis was performed at 50–70 V for 3 h.

### 2.6 Immunoblot analysis

The protein samples obtained from the 2DE gels were transferred onto PVDF membranes, which were previously activated with 100% methanol to enable the optimal transfer of proteins. The membrane was treated with a blocking solution containing 5% skim milk in PBS with 0.1% Tween 20 (PBST) for 2 h at RT, followed by three 5-min washes to minimize non-specific binding. Subsequently, the membranes were incubated at RT for 1 h with a 1:100 dilution of pooled sera obtained either from a normal mouse or 10^6^ tachyzoites of *T. gondii* RH-infected mice, which were collected 5 days post-infection and prepared in 5% BSA. After washing three times with PBST, the blots were incubated with the corresponding goat anti-mouse IgM (heavy chain) secondary antibody, HRP (Thermo Fisher 62-6820), and diluted 1:5000 in blocking buffer for 1 h at RT. The membranes were washed five times before detection using Clarity Western ECL Substrate (Bio-Rad Cat# 170-5060).

### 2.7 Determining specific proteins using liquid chromatography/tandem mass spectrometry (LC/MS–MS)

Consistent spots containing the aligned immunoreactive proteins that detected only in *T. gondii*-infected mouse sera were excised from the five 2DE gels of tachyzoite samples of IPG strip pH3-10 and sent to the LC/MS–MS service for analysis.

### 2.8 *In silico* analysis of the novel biomarker

To characterize the antigenicity of the proteins, the Linear B cell epitopes of the predicted proteins were determined using ABCprep, BCPreds, and BepiPred-2.0, while the peptides expected to be subjected to T-cell epitope processing and bind to MHC class I and II molecules were analyzed using the EIDB analysis resource tool.^1^ The 3D structure was generated using the I-TASSER server, and the 3D structure was modeled and visualized using the PyMOL molecular graphics system.

### 2.9 Expression and purification of recombinant proteins

DNA fragments of three discovered proteins of *T. gondii*, EF1γ, PGKI, and GAP50, with lengths of 1,182 bp, 1,248 bp, and 1,293 bp, respectively, were sub-cloned into the plasmid pGEM-T Easy Vector (A1360, Promega, Madison, WI, United States) before being cloned into the pET21b (+) plasmid vector. Next, the recombinant plasmid encoding *T. gondii* EF1γ was transformed into *Escherichia coli* BL21 DE3 PlysS, while the plasmid DNAs harboring *T. gondii* PGKI and GAP50 were transduced into the *E. coli* BL21 DE3 strain, followed by induction with 1 mM isopropyl-b-D-thiogalactopyranoside (IPTG) (Sigma). PGKI was mainly expressed as soluble form in PBS (pH 7.4) lysis buffer. EF1γ and GAP50 proteins were predominantly expressed as inclusion bodies (IB), and then, the protein pellet forms were solubilized and refolded according to previously reported methods (Nguyen et al., 2019). In summary, the induced culture was centrifuged to isolate the cell pellet containing the protein target inclusion bodies. The pellet was washed with Buffer A [10 mM Tris–HCl (pH 7.5)/10 mM EDTA/100 mM NaCl] and resuspended in Buffer A containing 1 mM freshly prepared PMSF. After sonication, the lysate was stirred for 1 h at 4°C with an equal volume of 8 M urea solution. Following centrifugation at 10,000 rpm by a A650TC Cryste rotor, the IB pellet was washed with Buffer B [10 mM Tris–HCl (pH 7.5)/1 mM EDTA/1 M NaCl] and distilled water. The IBs were then re-suspended in solubilization buffer, [100 mM Tris–HCl (pH 7.5)/0.2 mM EDTA/6 M GuHCl], stirred on a magnetic stirrer for 2 h at room temperature (RT), and clarified by centrifugation for 1 h at 4°C. The supernatant containing the solubilized IBs was collected by centrifugation at 17,000 rpm by a Labogene 1730R machine, and then, the refolding was initiated by rapid dilution of the denatured IBs in freshly prepared refolding buffer (100 mM Tris–HCl/0.5 M L-arginine/0.2 mM EDTA, pH 7.5) and incubated for 36–48 h at 10°C. The refolded preparation was dialyzed against 20 mM phosphate buffer (pH 7.5), containing 300 mM NaCl, 10 mM imidazole, and 100 mM freshly prepared urea for 48 h, with buffer changes every 12 h. The dialyzed sample was centrifuged at 10,000 rpm and clarified further by filtration through a 0.45 μm membrane. Finally, batch purification on Ni–NTA resin was conducted for the His-tagged three recombinant proteins according to the manufacturer’s instructions, followed by concentration using a Centricon filter unit.

Western blotting analysis was performed to confirm protein expression. The purified proteins were loaded onto a 10% SDS-PAGE gel and then transferred onto activated PVDF membranes. A blocking buffer (5% non-fat milk in PBS) was used to block the membrane for 2 h at RT. After washing the membrane with PBS-T (PBS containing 0.1% Tween 20), the first anti-mouse 6× His-tag antibody (dilution 1:10000) was incubated with the membrane in a blocking buffer for 1 h at RT. The membranes were then washed three times with PBS-T and incubated with the secondary goat anti-mouse antibody conjugated with HRP (Ab97046, Abcam) in blocking buffer and diluted (1,30,000) for 45 min at RT. The protein bands were visualized using a Bio-Rad ChemiDoc XRS+.

### 2.10 Immunoblot analysis of rAgs in sera from mice infected with *Toxoplasma gondii*

The purified *T. gondii* proteins such as EF1γ, PGKI, and GAP50, in comparison to BSA, along with TLA of *T. gondii* RH tachyzoites in uninfected APRE19 cells as a reference, were separated on a 10% SDS-PAGE gel with 20 μg/lane. Afterward, the proteins were transferred onto activated PVDF membranes and then blocked with 5% skim milk at RT for 2 h. Following three washes with PBS-T, 10^6^ *T. gondii* tachyzoites of mouse sera collected at 5 dpi (dilution 1:100 in 5% BSA) were used as the primary antibody, whereas normal mouse sera served as the control, and the membranes were incubated for 1 h at RT. The membrane was then washed again three times with PBS-T and incubated with goat anti-mouse IgM (heavy chain) secondary antibody HRP (Thermo Fisher 62-6820) and diluted 1:3000 in 5% BSA for 1 h at RT. Finally, the protein bands were visualized as described previously.

### 2.11 Production of europium nanoparticle (Eu NP) conjugates

To generate complexes of Eu NP and antibody, PS-COOH Eu NP beads with a size of 0.2 μm (#FCEU002, Bangs Lab) were conjugated with either goat anti-mouse IgM antibody (ARG21517, Arigo Biolaboratories) or goat anti-human IgM antibody (K0211481, KOMABIOTECH). Initially, 20 μL of Eu NPs was added to 754 μL of 0.05 M MES buffer (pH 6.1, Sigma–Aldrich) and then agitated for 1 h at 25°C in the presence of 26 μL of 5 mM EDC (Thermo #22980) and 200 μL of 50 mM sulfo-NHS (Sigma #56485-1G) to activate the COOH groups present on the surface of Eu NPs. The surplus EDC and sulfo-NHS were removed by subjecting the mixture to centrifugation at 27,237×*g* for 10 min at 4°C. Subsequently, the activated Eu NPs were combined with 60 μL of 1 mg/mL antibody in 940 μL of 0.1 M sodium phosphate at pH 8. This mixture was allowed to react for 2 h at 25°C. Following centrifugation at 27,237×*g* for 10 min, the precipitated bioconjugates were collected, washed once, and resuspended in 400 μL of 2 mM borax pH 9.0 containing 0.1% BSA. Finally, the bioconjugates were stored in the dark at 4°C for subsequent use (Duong et al., 2022).

### 2.12 Rapid FICT

To establish immunochromatographic test strips, the NC membrane (10 μM) was coated with antigen or antibody using the BioDot Dispenser machine (BioDot, California, US). For standardizing the lateral flow reaction, the membrane was coated with 0.05 mg/mL of rabbit anti-Goat IgG (H + L) antibody (ARG21945, Arigo Biolaboratories) to serve as the control line (CL). The test line (TL) was coated with 0.3 mg/mL purified rAg *T. gondii*-GAP50 to detect IgM in the serum of patients with toxoplasmosis. The conjugate, sample, and absorbent pads were then attached to a backing card to complete the process.

The strip was used for FICT after drying at 30°C for 2 days. In summary, 6 μL of Eu NP conjugated either with anti-mouse IgM or anti-human IgM was placed onto the conjugate pad. Then, a mixture of either mouse or human serum in 75 μL of distilled water (DW) was thoroughly diluted into 125 μL of diluent buffer (0.1 M Tris pH 9, 0.1% gelatin, and 0.5% Tween 20) and then applied to the sample pad. After 15–20 min, a portable fluorescent strip reader (MEDISENSOR, Daegu, Korea) was used to interpret the results at excitation and emission wavelengths of 365 and 610 nm, respectively. The TL/CL ratio was used to calculate the quantitative diagnostic parameters of the FICT.

### 2.13 Determination of the FICT cutoff value and the limit of detection (LOD)

The cutoff value of the FICT was determined by calculating the mean of the normal sera (*n* = 10) plus three times the standard deviation (SD) using the TL/CL value. Subsequently, to establish the LOD (Armbruster and Pry, 2008) for the FICT test involving the coating rAg Tg-GAP50, standard *T. gondii*-infected human sera (code 13/132) were prepared by spiking 10 μL of normal sera, which were then subjected to the FICT.

### 2.14 Statistical analysis

All graphs were generated using GraphPad Prism (version 9.0, La Jolla, CA, USA) and presented as the mean and standard deviations (SD) of biological replicates. One-way and two-way analysis of variance (ANOVA) were used to analyze the ELISA and FICT.

## 3 Results

### 3.1 *In vitro* culture of *Toxoplasma gondii* tachyzoites and collection of sera from infected mice

To obtain highly pure protein samples, tachyzoites were mass-cultivated with APRE19 cells and subsequently purified to ensure no contamination, as shown in Supplementary Figures S1A,B.

**Figure 1:**
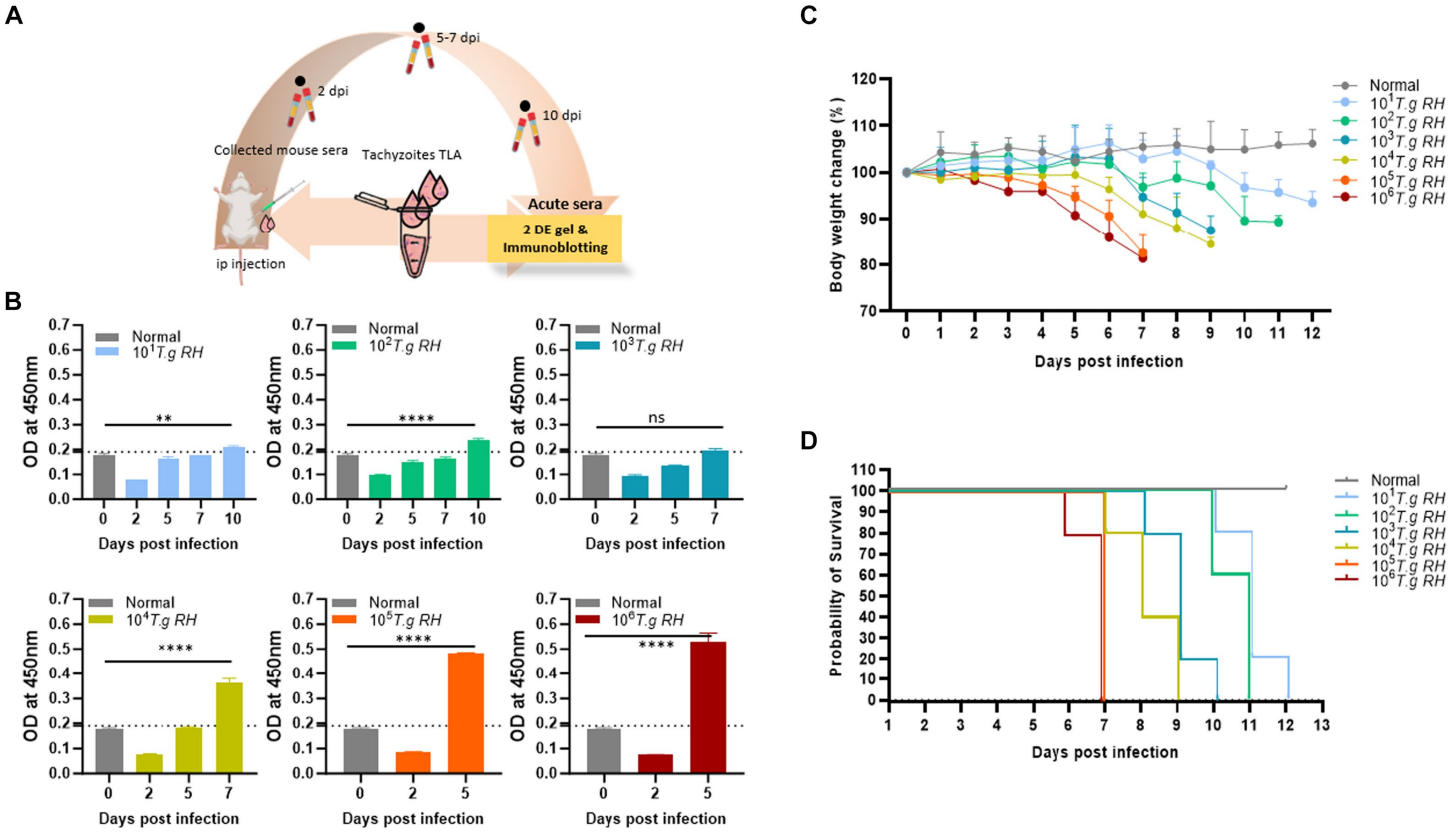
Collection of sera from mice infected with *T. gondii*. **(A)** Scheme of the animal model: pure tachyzoites of *T. gondii* RH strain were intraperitoneally (i.p.) injected into the mouse belly, and blood was collected at 2–10 days post-infection. **(B)** TLA-based ELISA determination of IgM in the sera of BALB/c mice immunized with different number of tachyzoite. The ELISA data (each group *n* = 3) was shown as means ± SD. The dot-line indicated the cutoff value of ELISA which determined as mean of normal sera plus three times of SD. **(C)** Body weight change and **(D)** survival rate of infected mouse groups (each group *n* = 5) monitored every day. Two-way analysis of variance (ANOVA) was used to analyze the ELISA. ns, not statistically significant; ^*^p < 0.05, ^**^p < 0.01, ^***^p < 0.001, and ^****^p < 0.0001.

Pure and fresh tachyzoites of the *T. gondii* RH strain were i.p. injected into the mouse belly, and blood was collected 2–10 days post-infection (Figure 1A). The RH strain of *T. gondii* was highly virulent in mice, resulting in the mortality of all mouse groups infected with tachyzoites ranging from 10^1^ to 10^6^ 12 days post-infection (Figures 1C,D).

During this study, mice inoculated with 10^5^ and 10^6^ tachyzoites were survived for only 7 dpi and lost 20% of their body weight before death. However, these two groups showed a strong IgM immunological response to the tachyzoite TLA base-indirect ELISA response at day 5 post-infection (Figure 1B).

The group of mice that received 10^4^ to 10^1^ tachyzoites persisted longer, until 9, 10, 11, and 12 dpi, and experienced an approximately 10–15% decrease in body weight before eventually succumbing to the infection; however, the IgM responses were observed to be comparatively lower when mice were infected with higher tachyzoite doses (Figure 1).

### 3.2 Electrophoresis of 2DE

To analyze the proteomics of *T. gondii* at tachyzoite stages, a comparative assessment was conducted between tachyzoite TLA and uninfected cells using 2DE gel, separating the proteins based on their isoelectric points (pIs) and molecular weights.

**Figure 2:**
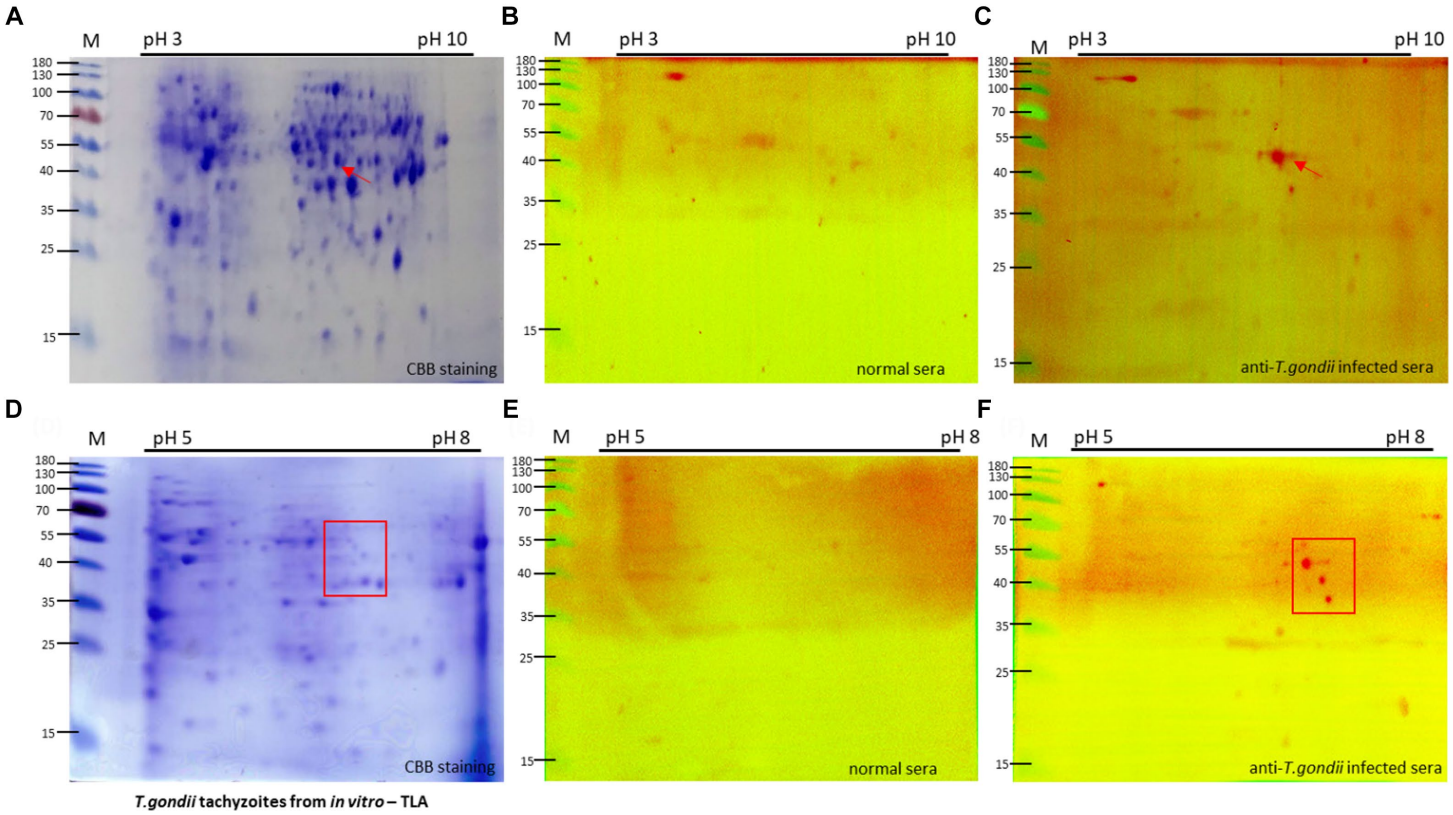
Two-dimension electrophoresis (2DE) and immunoblot analysis of tachyzoite total lysate antigen (TLA). 2DE of tachyzoites was conducted using IPG strip NL pH3-10 **(A)** and pH 5–8 **(D)** for analysis of the proteomics of *T. gondii*. Specific protein spots, indicated by red arrows, exhibited differential expression in tachyzoites compared with uninfected cells (Supplementary Figure S2). TLA proteins of tachyzoites **(A,D)** were transferred to a PVDF membrane and then normal sera **(B,E)** in comparison with *T. gondii*-infected mouse sera **(C,F)** diluted 1:100 were probed, followed by detection of anti-mouse IgM-HRP antibodies.

Using the data obtained from the 2DE of the IPG strip with a pH range of 3–10, distinct differences were observed in the tachyzoite sample (Figure 2A) compared with uninfected cells (Supplementary Figure S2). This indicated major variations in protein expression and composition primarily in the pH range of 5–8 area.

To specifically target the antigen identified by IgM antibodies, a group of mice infected with 10^5^ and 10^6^ tachyzoites, known to exhibit an intense IgM immune response, was selected for Western blotting analysis of tachyzoite TLA in 2DE. Compared with the sera from normal mice, the sera of *T. gondii*-infected mice exhibited extensive reactivity of IgM antibodies toward protein spots within the 40–55 kDa range at pH 5–8 (Figures 2B,C).

To investigate these proteins more thoroughly, Western blotting analysis of tachyzoite TLA in 2DE probed with mouse serum was conducted using IPG strips with a narrow pH range of 5–8, maintaining that the spots within the 40–55 kDa range at approximately pH 7 were consistently visible (Figures 2D–F).

The same spot pattern was consistently observed in the Western blotting assessment of the TLA of tachyzoites in 2DE probed with serum from mice via IPG strips at pH 3–10 in triplicate (Supplementary Figure S3) and pH 5–8 (Supplementary Figure S4).

### 3.3 Determining the presence of certain proteins by incorporating LC/MS–MS

To get insights into the composition and identification and characterization of molecules based on their mass-to-charge ratio through MS analysis, the spots in question were revealed to be *T. gondii* EF1γ (44 kDa at pI 5.97), PGKI (44.6 kDa at pI 6.57), and GAP50 (46.6 kDa at pI 6.46), with high percentages of peptide definition rates (Table 1).

**Table 1:** Protein identification analysis using LC–MS/MS.

*N*	% Coverage	Accession #	Name	Species	Peptides (95%)	Sequences	Remark	Kda	pI
1	74.9	KAF4638113.1	Putative elongation factor 1-gamma [*Toxoplasma gondii*]	*Toxoplasma gondii*	64	MK**LLTPK**DDVRGR**KVQLVAAFLDLPLQTVPFTVGKDDKDPAFLAK**SPLGR**LPLLESEVGGVCLFESNAICR**FLARLRADKCLYGETLAEQGQVDMWLDFSTLEVEIPMCCLVQGGKVAER**AQSDLAQALNAVDAHLK**TRTFMVGENITIADLCLVAVLSYGFR**SGKVDAAALLEK**RPYLK**RFYETVVNQK**SFK**KIFGEAKAAPQAAAK**KETPK**AAAKPAQSAGDDEEPAK**KPAVK**CELDLLPEPTMDLNEWKR**VYSNTK**DLYGTAMKWFWEHLDAAGYSLWYMK**YQK**LEGECTVAFVTSNQLGGFLQR**IDPAFRKYSFGVVDVMGENGCFDIEGVWLFR**GQDVPSLMKDHPSYEYHTWQKLDVASAKDKQLVADFWCACDDIQGRPIADSK**VWK	Best ID (intact protein form)	44	5.97
2	63.3	KAF4645014.1	Phosphoglycerate kinase PGKI [*Toxoplasma gondii*]	*Toxoplasma gondii*	22	MLANK**LGIQDVGAQLTGK**SVLIR**VDFNVPMKDGVVQDATR**IK**ATLPTLEYALSK**NPRCLVLLSHAGRPDGRVQMK**YTLKPVAAALQEFLPKKVTFVEDCVGPKAEEAVQAAKNGEILVMENVR**FHIEEEGKGVDEQGNKIK**ASPEAIAK**FKADLTK**LGDIYINDAFGTAHRAHASMVGIEVPVRAAGLLMK**KELDYFSK**ALECPEKPFLAILGGAK**VRDK**IQLIENMLDR**VQMMIVAGGMAFTFKKVLDNMPIGDSLFDEEGAKIVPDIMAKAKAKGVEMLFSCDFLCADKFDNNANTQICEDKTGIPDGWMGVDIGPKTVEK**ATEMILR**AKTLVWNGPPGVFEMSNFAK**GSIAFCGAVAK**ATEK**GCITIVGGGDTAALVER**EGYASK**VSHVSTGGGASLELLEGKTLPGVAALSNK**	2nd ID (minor protein)	44.6	6.57
3	49	KAF4638216.1	Acid phosphatase GAP50 [*Toxoplasma gondii*]	*Toxoplasma gondii*	20	MAGAPVAAAPAASSRVAGDSGRSRALSSFFSVFSLCVFSVFLATPTAVVAQLK**FVGLGNWGSGSYGQKTVADTLKK**VAANEHISFIASPGSNFLGGVSSLNDTR**WQSEFENVYSDANGALKMPFFTVLGVDDWSR**NYTSEALR**TELTYAVTSEQIKDGKLAPADATEAAAAENHGYPK**WTLPNWWYHYLMHFPANTGGAFINSGHKDMSVGMIFIDTWVLSSSFPFSNVTSR**AWADLEKTLELAPKILDYIIVVADRAVYSSGASKGDSMLQYYLQPLLK**KANVDAYISGYDFSLEVISDDNISHVSCGAGSK**AAGSPIVK**HSGSLYYAGETGFCLFELTAEGLVTR**LVSGTTGETLYTHK**QPLKNRPER**KSIDAFNFVSQLPEVRYYPVPEMGK**MPGRDVFVRVVGTIGLCIATIFLSLSVANGLSRYMK	3rd ID (minor protein)	46.6	6.46

Confidence: Green (>95%) > Yellow (>1.0%) > Red (< 0.1%); Gray: not detected.

In particular, EF1γ had coverage of 74.9, and 64% of the identified peptides corresponded to this protein, while PGKI and GAP50 exhibited coverage of 63.3 and 49%, respectively, of these sequences, and 22% of the recorded peptides were similar to them (Table 1).

### 3.4 Prediction of epitopes in recombinant antigens

The sequences of *T. gondii* EF1γ, PGKI, and GAP50 were evaluated using bioinformatics to predict linear epitopes using three different prediction programs: (ABCpred (threshold: 0.8), BCPreds (specificity: 75%), and [IEDB]-BepiPred (threshold 0.5) (Supplementary Figure S4). A total of 19 (threshold: 0.8), 8 (specificity: 75%), and 12 (threshold: 0.5) linear epitopes were predicted via ABCpred, BCPreds server 1.0, and IEDB-BepiPred, respectively, for EF1γ. PGKI was found to possess 17, 4, and 14 linear epitopes, respectively, and GAP50 comprised 15, 7, and 11 linear epitopes, respectively (Supplementary Figure S5).

**Figure 3:**
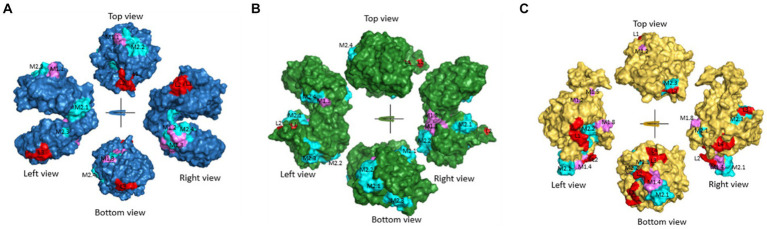
Predicted common linear epitopes and potential MHC-I and MHC-II binding peptide sequences. **(A)** EF1γ, **(B)** PGKI, and **(C)** GAP50 were analyzed and presented as 3D modeling. Red indicates common linear epitopes and purple and cyan indicate MHC I and II, respectively.

In the case of EF1γ, the predictions from the three programs indicated the presence of GK (L1) at positions 165–166, AAAKKET (L2) at positions 205–211, and DVASAKD (L3) at positions 362–368. For PGKI, D (L1) at position 69 and G (L2) at position 137 were consistent across all three prediction programs. Similarly, GAP50 exhibited common epitopes, including SRVAGDSGR (L1) at positions 14–22, SGH (L2) at positions 203–205, SGASKGD (L3) at positions 262–268, and QPLKN at positions 362–366 (Table 2). These predicted common linear epitope sequences of EF1γ, PGKI, and GAP50 were analyzed and presented as 3D modeling (Figure 3).

**Table 2:** Potential linear epitopes.

	Linear epitopes		
		Position	Sequence
Tg-EF1γ	L1	165–166	GK
	LE2	205–211	AAAKKET
	L3	362–368	DVASAKD
	Coverage (%)	16/394	4.06%
Tg-PGKI	L1	69	D
	L2	137	G
	Coverage (%)	2/416	0.48%
Tg-GAP50	L1	14–22	SRVAGDSGR
	L2	203–205	SGH
	L3	262–268	SGASKGD
	L4	362–366	QPLKN
	Coverage (%)	24/431	5.57%

In addition, Figure 3, Tables 2, 3 highlighted the specific peptides associated with T-cell epitope processing, and MHC binding of *T. gondii* EF1γ, PGKI, and GAP50, respectively, is reliable between two out of three predictor systems.

**Table 3:** Potential MHC-I and MHC-II binding peptide sequences.

	MHC-I binding peptide			MHC-II binding peptide		
		Position	Sequence		Position	Sequence
Tg-EF1γ	M1.1	188–194	NQKSFKK	M2.1	39–53	DPAFLAKSPLGRLPL
	M1.2	308–316	RIDPAFRKY	M2.2	181–190	RFYETVVNQK
	M1.3	346–353	LMKDHPSY	M2.3	245	T
				M2.4	301–314	QLGGFLQRIDPAFR
	Coverage (%)	24/394	6.09%	Coverage (%)	40/394	10.1%
Tg-PGKI	M1.1	40–48	RIKATLPTL	M2.1	16–35	TGKSVLIRVDFNVPMKDGVV
	M1.2	185–192	AAGLLMKK	M2.2	47–62	TLEYALSKNPRCLVLL
	M1.3	216–224	KVRDKIQLI	M2.3	146–156	AIAKFKADLTK
				M2.4	206–221	KPFLAILGGAKVRDKI
	Coverage (%)	26/416	6.25%	Coverage (%)	63/416	15.1%
Tg-GAP50	M1.1	105	W	M2.1	36–50	CVFSVFLATPTAVVA
	M1.2	140–148	EALRTELTY	M2.2	181–193	LPNWWYHYLMHFP
	M1.3	180–184	TLPNW	M2.3	246–265	KILDYIIVVADRAVYSSGAS
	M1.4	192–200	FPANTGGAF			
	M1.5	226	F			
	M1.6	246–254	KILDYIIVV			
	M1.7	257–260	RAVY			
	M1.8	380–382	SQL			
	Coverage (%)	41/431	9.51%	Coverage (%)	48/431	11.1%

The linear epitopes of GAP50 were mostly located on the outer surface, covering 5.57% of the sequence (see Table 2), representing the highest coverage compared with other types in the 3D model. In contrast, the linear epitopes of EF1γ and PGKI exhibited lower coverage, with 4.06 and 0.48% of the sequence, respectively. Regarding MHC-binding peptides, EF1γ, PGKI, and GAP50 exhibited total coverage percentages of 16.19, 21.35, and 20.61% of their respective sequences (Table 3). Notably, those of GAP50 were mainly exposed on the surface, while those of PGKI were hidden rather than surface-exposed. As a result, GAP50 seems to efficiently present strong epitopes and MHC-binding peptides on the surface compared with the others (Figure 3).

### 3.5 Expression of *Toxoplasma gondii* EF1γ, PGKI, and GAP50 recombinant proteins

**Figure 4:**
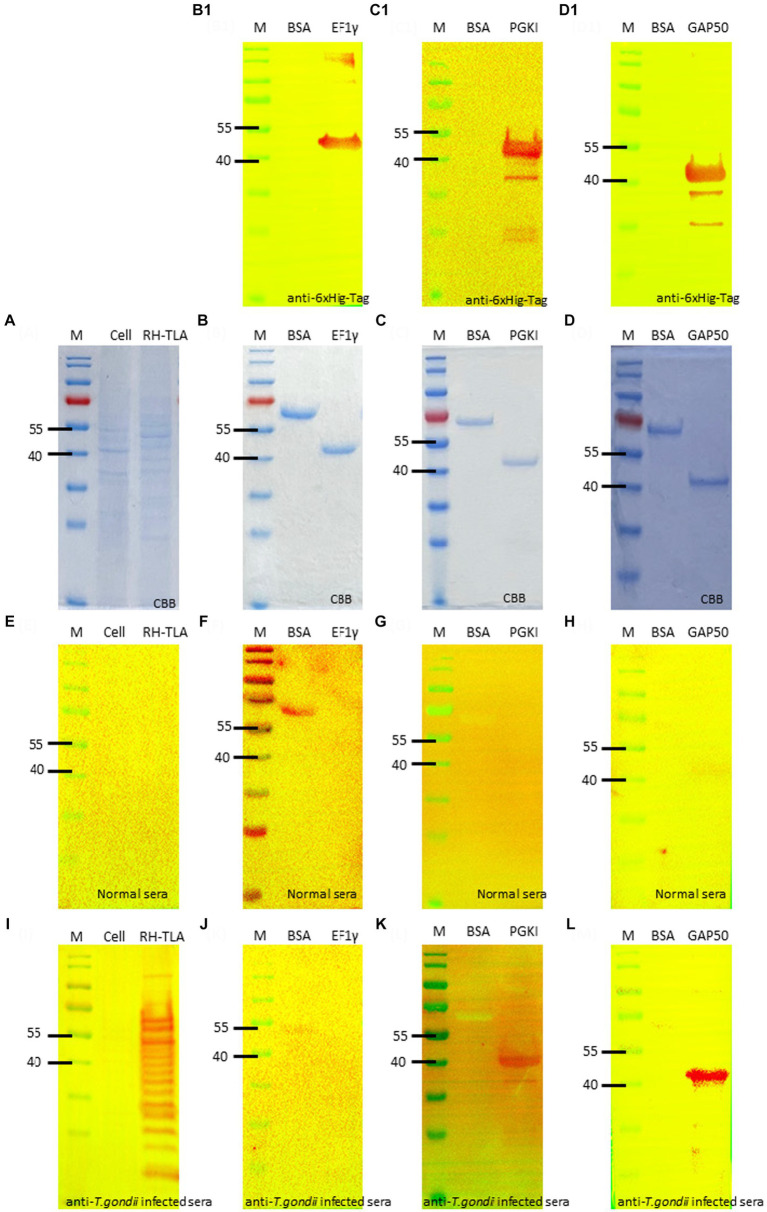
SDS-PAGE and Western blotting. **(A)** SDS-PAGE of *T.gondii* TLA in comparison with uninfected cell total lysate. Western blotting using anti-6× Hig-Tag to confirm the expression of the purified recombinant antigen **(B,B1)** EF1γ – 46 kDa; **(C,C1)** PGKI—48.5 kDa, and **(D,D1)** GAP50–50 kDa in comparison with BSA as the negative antigen. Western blotting to detect IgM antibodies probed by **(E–H)** normal mouse sera and **(I–L)** *T. gondii* 10^6^ tachyzoite-infected mouse sera as the primary antibody of **(E,I)** of tachyzoites TLA in comparison with uninfected cells; **(F,J)** rAg EF1γ; **(G,K)** PGKI, and **(H,L)** GAP50 in comparison with BSA. Protein was run with 20 μg/lane. Anti-6**×** Hig-Tag mouse IgG-HRP was diluted 1:10,000 in 5% non-fat milk. Goat anti-mouse IgM (heavy chain)-HRP was diluted 1:3000 in 5% BSA. A total of 10^6^ *T. gondii* tachzyoites of RH strain-infected mouse sera collected on day 5 post-infection were used. M, Marker PageRuler Prestained Protein Ladder (#26617-Thermo Scientific).

The full-length coding sequence of the EF1γ, PGKI, and GAP50 genes was cloned into expression vector pET21b (+) to produce a final construct. The purified recombinant antigens, fused with 6× His-tag, formed proteins of 46, 48.5, and 50 kDa, respectively, for EF1γ (Figures 4B,B1), PGKI (Figures 4C,C1), and GAP50 (Figures 4D,D1), which were confirmed by the presence of an intense band of approximately 40–55 kDa on SDS-PAGE and Western blotting probed with the anti-6× Hig-Tag antibody. Circular dichroism (CD) spectroscopy revealed that three recombinant proteins have α-helical structures (Supplementary Figure S6).

To verify the detectability of *Toxoplasma* IgM antibodies against recombinant antigens, Western blotting assays were performed using normal mouse sera and *T. gondii*-infected mouse sera as primary antibodies. The recombinant antigens EF1γ, PGKI, and GAP50 were compared with BSA, *T. gondii* tachyzoite TLA, and uninfected cells (Figures 4A,E,I). Figures 4B,F,J show that EF1γ was not recognized by IgM of *T. gondii*-infected mouse sera, whereas PGKI (Figures 4C,G,K) and GAP50 (Figures 4D,H,L) were extensively detected (Supplementary Figure S7).

Subsequently, PGKI and GAP50 were subjected to ELISA using mouse and human serum samples. The cross-reactivity with *P. vivax* and regular human serum was difficult to eliminate when PGKI was used as antigen coating (Supplementary Figure S8).

### 3.6 Rapid FICT

**Figure 5:**
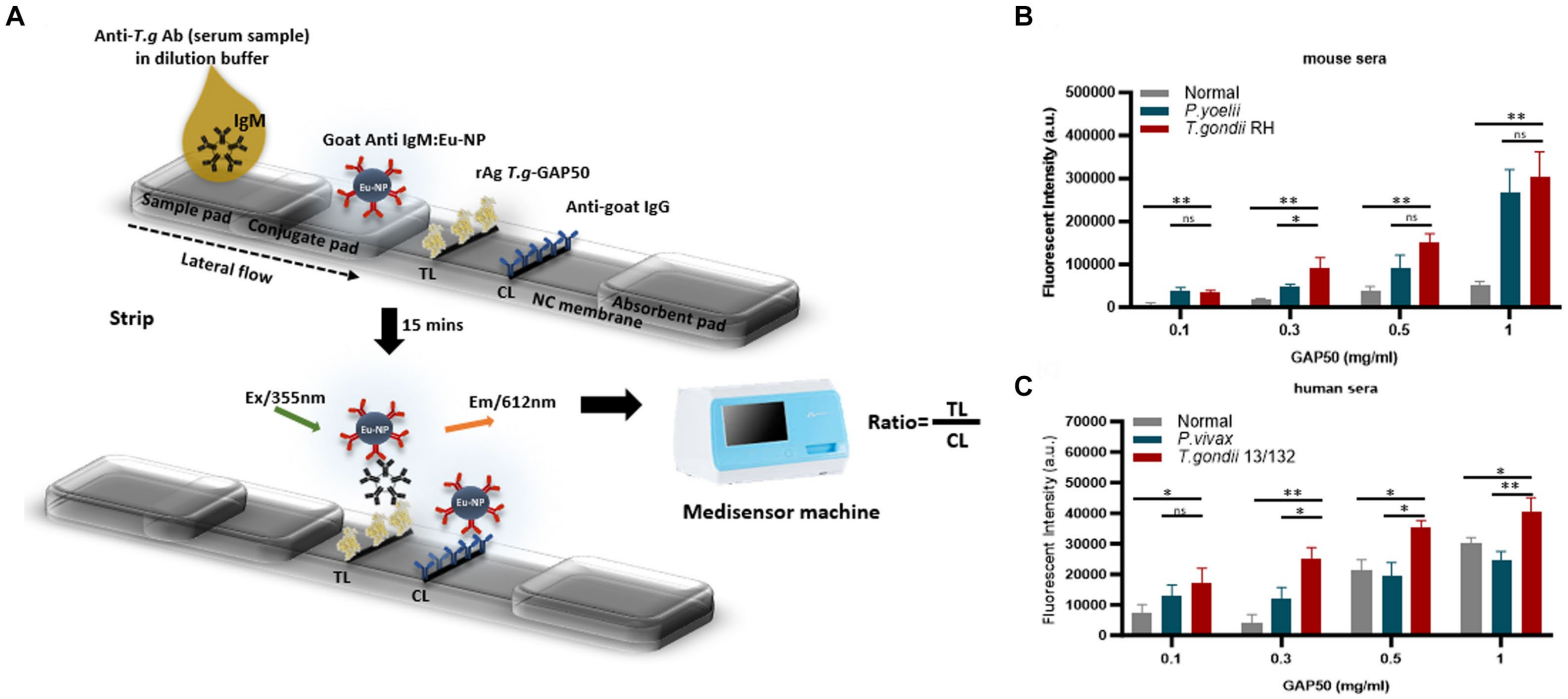
Strip test diagram and an optimization coating strip. **(A)** Strip test diagram: The NC membrane was coated with 0.05 mg/mL of rabbit anti-goat IgG (H + L) as a control line (CL). The test line (TL) was coated with 0.3 mg/mL purified rAg *T. gondii*-GAP50 to detect IgM in patients with toxoplasmosis. The conjugate, sample, and absorbent pads were then attached to a backing card to complete the process. Overall, 6 μL of Eu NP-conjugated either with anti-mouse IgM or anti-human IgM was placed onto the conjugate pad. Then, a mixture of either mouse or human serum in 75 μL of distilled water (DW) was thoroughly diluted into 125 μL of diluent buffer and then applied to the sample pad. After 15–20 min, a portable fluorescent strip reader was used to interpret the results at excitation and emission wavelengths of 365 and 610 nm, respectively. The TL/CL ratio was used to calculate the quantitative diagnostic parameters of the FICT. **(B,C)** Optimization of the rAg concentration coating strip for the FICT to detect IgM in mouse and human sera. The NC membrane was coated with 0.1, 0.3, 0.5, or 1 mg/mL of purified rAg Tg-GAP50 as the TL. Mouse **(B)** and human sera **(C)** were analyzed by FICT using Eu-NP-conjugated anti-mouse IgM and/or anti-human IgM. The interaction of rAg with Abs present in sera was determined by measuring the fluorescence intensity (365 nm excitation and 610 nm emission). The rAg Tg-GAP50 coating was used to test cross-reactivity to *P. vivax* and *P. yoelii*. In total, 2 μL of serum was used per reaction. The TL fluorescent (*n* = 3) was shown as mean ± SD. Two-way analysis of variance (ANOVA) was used to analyze the FICT. ns: not statistically significant, *p < 0.05, **p < 0.01, ***p < 0.001, and ****p < 0.0001.

To further investigate the capacity of strip testing to detect antibodies against IgM in serum samples, purified rAg GAP50 was coated at a variety of concentrations (0.1, 0.3, 0.5, and 1 mg/mL) as a single TL on NC membranes. An immunochromatographic test strip (Figure 5A) utilizing Eu NP-conjugated anti-mouse IgM (diluted 80-fold) (Figure 5B) and/or anti-human IgM (diluted 320-fold) (Figure 5C) was used to detect mouse and human sera, respectively. With GAP50 coated at 0.3 mg/mL on the NC membrane, it was possible to reduce the non-specific fluorescence intensity when examining cross-interactions involving *P. vivax*, *P. yoelii*, or typical serum (raw data are shown in Supplementary Figure S9).

Furthermore, an FICT included the test line coated with 0.3 mg/mL GAP50 and the control line coated with 0.05 mg/mL rabbit anti-goat IgG (anti-gIgG) to quantify the conjugate required in each reaction.

**Figure 6:**
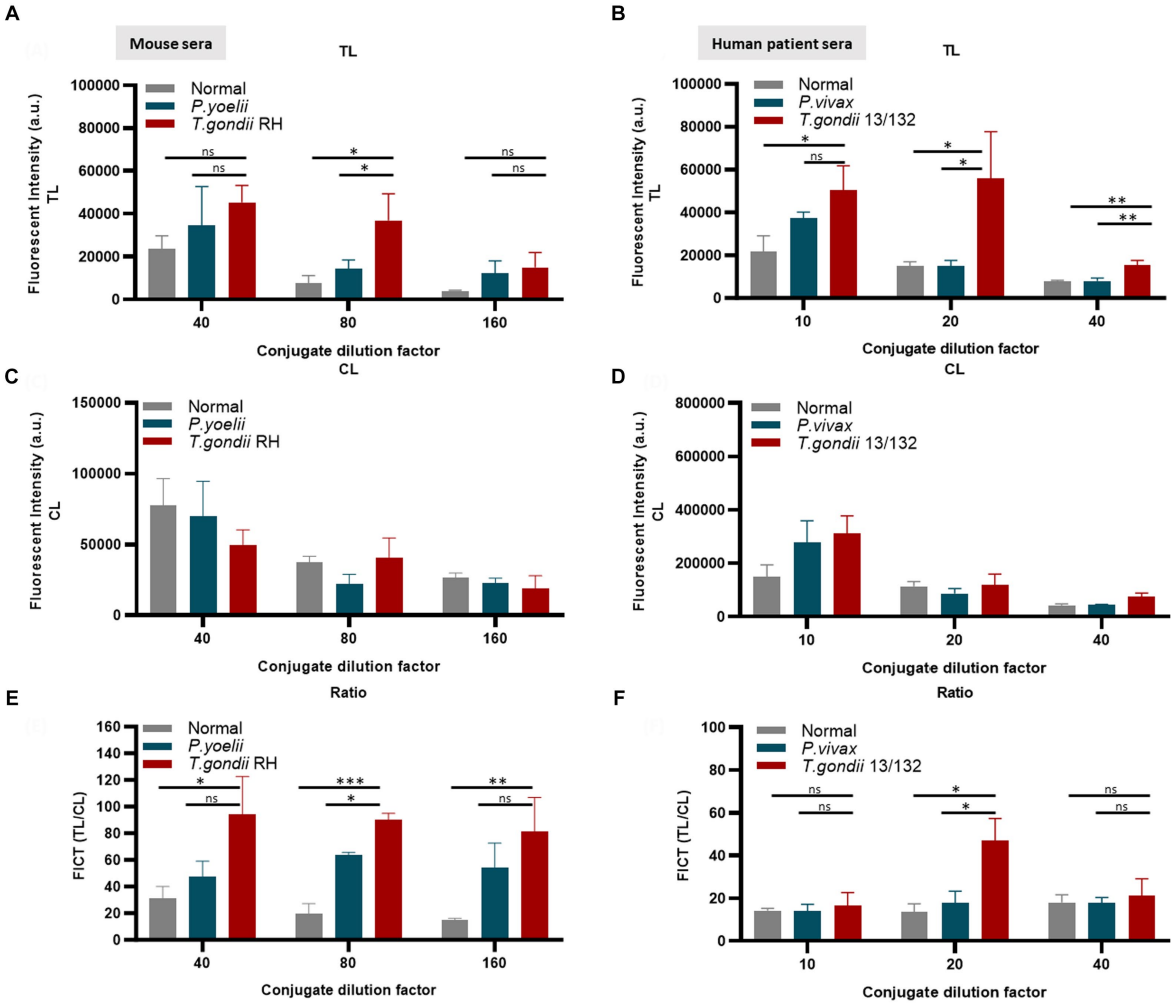
Evaluation of the quantity of conjugate required in each reaction of FICT to detect IgM. An immunochromatographic test strip included the test line (TL) coated with 0.3 mg/mL rAg Tg-GAP50 and the control line (CL) coated with 0.05 mg/mL rapid anti-goat IgG (anti-gIgG). EuNP-conjugated anti-mouse IgM **(A,C,E)** or conjugated anti-human IgM **(B,D,F)** was dropped onto the conjugate pad, and the strip was dipped in a mixture of sera for 15 min. A portable fluorescence detector (excitation at 365 nm and emission at 610 nm) was used to measure the fluorescence signals of TL **(A,B)** and CL **(C,D)**. TL/CL values were used to determine the quantitative diagnostic value of the FICT **(E,F)**. Overall, 2 μL of serum was used per reaction. The fluorescent density of TL, CL, and ratio of TL/CL data (*n* = 3) was shown as means ± SD. Two-way analysis of variance (ANOVA) was used to analyze the FICT. ns, not statistically significant; ^*^p < 0.05, ^**^p < 0.01, ^***^p < 0.001, and ^****^p < 0.0001.

The application of conjugated anti-mouse IgM (diluted 80-fold) (Figures 6A,C,E) and/or anti-human IgM (diluted 20-fold) (Figures 6B,D,F) efficiently minimized non-specific binding at the TL associated with *Plasmodium* or normal serum while maintaining an appropriate TL/CL ratio (raw data are shown in Supplementary Figure S10).

### 3.7 Determination of the FICT cutoff value and LOD

Supplementary Figure S11 reveals that the ideal volume of human sera per reaction for FICT was 2 μL when various quantities (1, 2, 4, 8, 10, and 15 μL) of sera were compared. In our system, the utilization of 1 μL sera (diluted 1:200) in 200 μL per reaction was insufficient to distinguish IgM in a standard *T. gondii* patient serum 13/132 from other samples, while an increase in 15 μL of sera (diluted 1:13.3) was unable to eliminate the non-specific reaction when measuring the TL area. The greatest fluorescent signal at the test line (TL) was noted when using 8 μL of sera (diluted 1:25). However, this also led to an overall increase in the intensity at the control line (CL), resulting in a less impressive TL/CL ratio compared with the sample from patients infected with *P. vivax*. Non-specific signals appeared at the test line (TL) when 4 μL of serum (diluted 1:50) was tested with *P. vivax* patient sera. The TL/CL ratio of standard T. gondii patient serum 13/132 was three and five times higher than that of P. vivax and normal serum, when 10 μL of sera were used per reaction. However, there were big differences in TL density or TL/CL ratios when only 2 μL of the sample was used. (Supplementary Figure S11). It aids in cost reduction and facilitates efficient human serum screening for various diseases and evaluation of diverse experimental conditions, a particularly crucial consideration when using rodent models to align with ethical and resource considerations.

**Figure 7:**
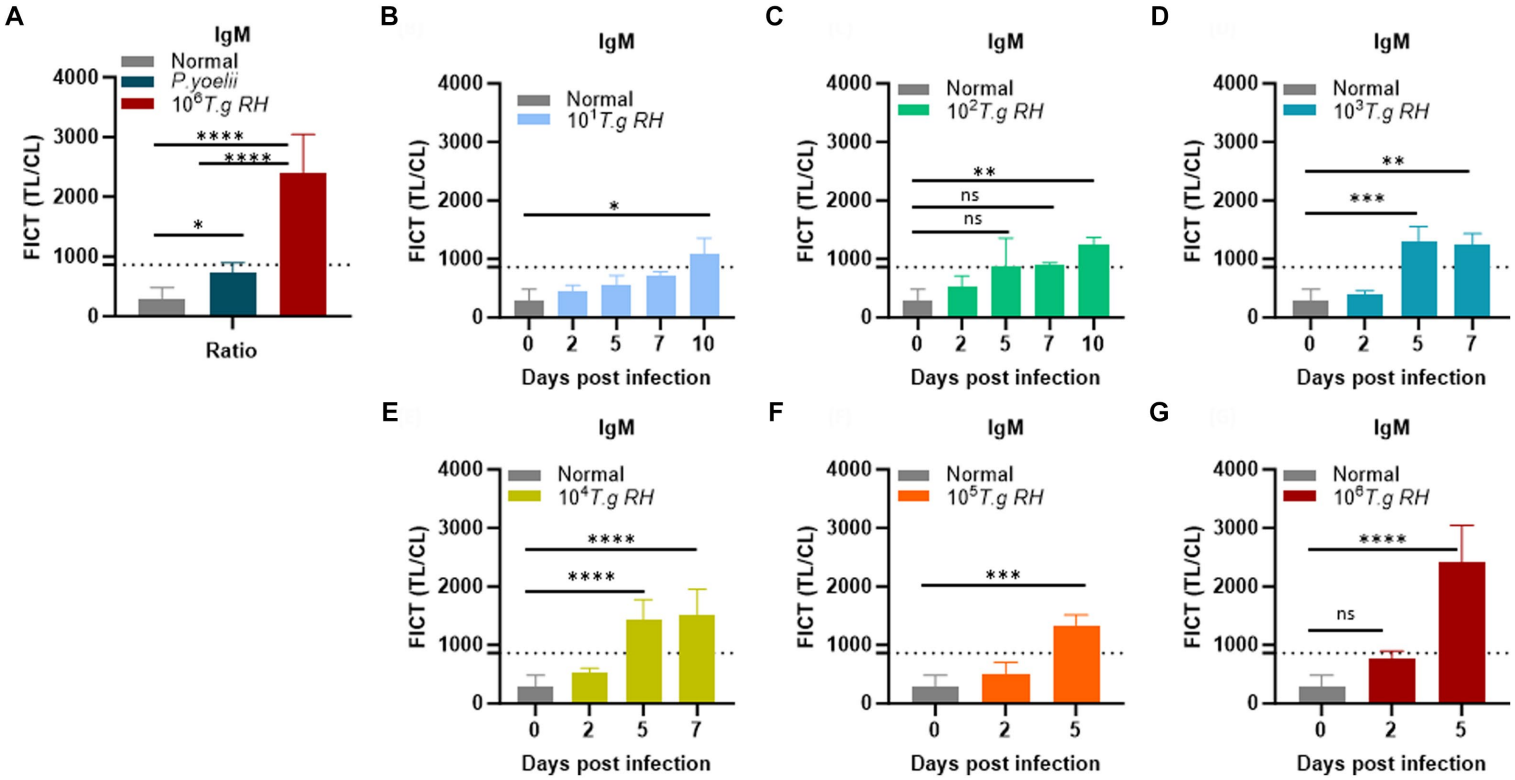
Determination of the FICT threshold value for mouse serum using the TL/CL ratio. **(A)** Normal; *P. yoelii* sera (each group, *n* = 10) and *T. gondii* infected 10^6^ tachyzoites at 5 dpi (*n* = 3) were shown with cutoff line. **(B–G)** *T. gondii* (10^1^–10^6^)-infected serum of BALB/c mice (each group, *n* = 3) at 2, 5, 7, and 10 dpi were subjected to the FICT. The cutoff value of the FICT was decided by calculating the mean of the normal sera (*n* = 10) plus three times the standard deviation (SD) using the TL/CL value when applying 2 μL of sera per strip reaction. Dotted line indicated that the cutoff value was 861.7 base on the TL/CL ratio. The ratio of TL/CL data was shown as means ± SD. Two-way analysis of variance (ANOVA) was used to analyze the FICT. ns, not statistically significant; ^*^p < 0.05, ^**^p < 0.01, ^***^p < 0.001, and ^****^p < 0.0001.

To establish the FICT threshold value, 2 μL of sera per strip reaction was applied. For mouse sera, the cutoff value was determined by calculating the mean of normal sera (*n* = 10) plus three times the standard deviation (SD) based on the TL/CL ratio (Figure 7A and Supplementary Figure S12). These data provide support for the tachyzoite TLA-based ELISA, indicating that sera at 2 dpi from mice infected with *T. gondii* ranging from 10^1^ to 10^6^ exhibited fluorescence levels similar to the background signal. However, on days 5 and 7 pi, sera from mice infected with *T. gondii* ranging from 10^3^ to 10^6^ showed significantly higher detectability. In contrast, sera from *T. gondii-*infected mice at densities of 10^1^ and 10^2^ only produced positive results on day 10 pi (Figures 7B–G and Supplementary Figure S13).

We conclude that the FICT approach using GAP50 was more sensitive than TLA-based ELISA for detecting IgM levels in sera from rodents infected with *T. gondii* at 5 dpi (10^3^, 10^4^) and 7 dpi (10^3^).

**Figure 8:**
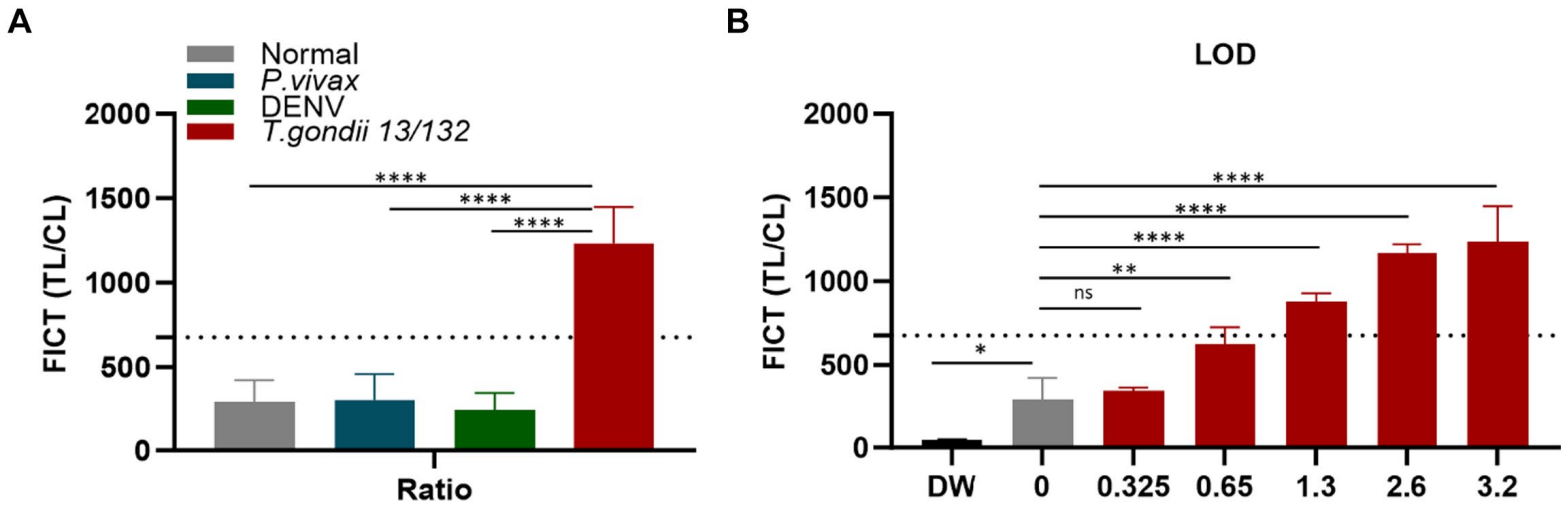
Determination of the FICT threshold value and Limit of Detection for human serum using the TL/CL ratio. **(A)** The cutoff value of FICT was decided by calculating the mean of normal human sera (*n* = 10) plus three times the standard deviation (SD) using the TL/CL value when applying 10 μL sera per strip reaction. Dotted line indicated that the cutoff value was 677.28 for distinguishing standard *T. gondii*-infected serum 13/132 (*n* = 1) with seronegative sera, *P. vivax*, and dengue sera (each group, *n* = 10). **(B)** *T. gondii* 13/132 were prepared by spiking in 10 μL of normal sera, which were then subjected to FICT. Thus, LOD for FICT using a coating of rAg Tg-GAP50 was established at 1.3 IU, equivalent to 4 μL tested. The ratio of TL/CL data was shown as means ± SD. Two-way analysis of variance (ANOVA) was used to analyze the ELISA and FICT. ns, not statistically significant; ^*^p < 0.05, ^**^p < 0.01, ^***^p < 0.001, and ^****^p < 0.0001.

In the context of FICT for human sera, the highest ideal volume of sera per reaction (10 μL of each sera per strip) (Supplementary Figure S11) was applied to determine the cutoff value. It similarly determined as 677.28 by calculating the mean of human seronegative sera (*n* = 10) plus three times the standard deviation (SD) based on the TL/CL ratio (Figure 8A and Supplementary Figure S14). Data demonstrate that the standard human *T. gondii* sera (code 13/132) (*n* = 1) significantly differ from that of seronegative sera and human cross-reaction controls (*n* = 20).

To establish the LOD for the GAP50-based FICT to human serum, a series of standard human *T. gondii* sera (code 13/132) with varying quantities (10, 8, 4, 2, and 1 μL equivalent to 3.2, 2.6, 1.3, 0.65, and 0.325 IU, respectively) were tested. In conclusion, positive results were observed up to 2 μL, which corresponded to 0.65 IU of anti-*T. gondii* antibodies (Figure 8B and Supplementary Figure S15).

Additionally, the OnSite commercial kit (REF: R0234CL) for IgM and IgG serum was employed to compare the results in parallel. According to the instructions of the kit, standard *T. gondii* 13/132 sera were tested in triplicate, with 10 μL utilized for each test, corresponding to 3.2 IU/strip. The outcome was visually inspected under the naked eye, with a faintly visible IgM-positive band (Supplementary Figure S16A). To assess the limit of detection (LOD) in parallel with FICT, the sera were similarly prepared by spiking them into 10 μL of normal sera and then analyzed in both the commercial strip and GAP50-FICT simultaneously. At each point of the LOD test, triplicated strips were evaluated. Inconsistent results were observed among them, while at least one strip exhibited a faint IgM band, not all strips showed the same result. This variation poses challenges in accurately determining the LOD when testing *T. gondii 13/132* by OnSite commercial kit (Supplementary Figure S16A). In addition, the OnSite commercial kits were evaluated by seronegative sera and human cross-reaction controls (Supplementary Figures S16B–D). The specificity was 96.77% (*n* = 30) with one Dengue sample showing cross-reactivity. However, as only one positive *T. gondii* sample was available, the sensitivity could not be determined.

These findings highlight the distinct immunoreactivity of GAP50, emphasizing its potential as a specific diagnostic biomarker to enhance the sensitivity of the FICT in detecting IgM in rodent samples. Additionally, novel GAP50 emerges as an attractive candidate antigen for integration in POCT for the IgM detection of *T. gondii* infections in patient samples.

## 4 Discussion

The commercial POC tests currently available in the market offer notable benefits, including their capacity to deliver immediate outcomes, affordability, and simplicity of use at onsite, and prolonged preservation period, collectively contributing to reducing the burden and impact of diseases on regions with limited resources. The majority of investigations have reported that *T. gondii* recombinant proteins can substitute natural tachyzoite antigen in IgG/IgM serological testing. The primary focus was the main surface antigen (SAG1). Sandwich ELISA utilizing purified SAG1 (P30) showed that all 37 acute toxoplasmosis patients exhibited significant IgM anti-P30 antibody levels (Santoro et al., 1985). Another research examined goat toxoplasmosis seroprevalence and found that the diagnostic rSAG1-ELISA had 92.66% sensitivity and 90.67% specificity (Bachan et al., 2018).

Another antigen of interest, known as GRA7 (dense granule antigen 7), exhibits a high abundance on the surface and within the cytosol of host cells, the lumen, and the membrane of the parasitophorous vacuole. GRA7 has been extensively studied since it elicits a strong immune response in both acute and chronic infections (Luo et al., 2019; Teimouri et al., 2019; Simon et al., 2020; Ybanez et al., 2020).

In addition, the full-length 65 kDa Matrix antigen 1-MAG1, predominantly localized in the matrix and wall of tissue cysts, is highly accurate and reliable indicator for detecting *T. gondii* IgG. The data obtained from the analysis of the mouse sera group and 105 patient human sera confirmed a sensitivity of 100 and 94.3%, respectively. However, the sensitivity for the assessment of IgM was found to be only 25.7% in the overall evaluation of the human sera. This is reinforced by the fact that MAG1 serves as a marker for bradyzoites while also being synthesized in tachyzoites (Gatkowska et al., 2015).

In a recent study (Ferra et al., 2020), based on the parasite surface receptor protein AMA1, which is correlated with host cell entry, ELISA assays exhibited a sensitivity of 99.4% for IgG and 80% for IgM when testing *T. gondii*-infected human sera. Furthermore, the utilization of AMA1 virus-like particles (VLPs) by other research groups also demonstrated over 90% sensitivity and specificity, highlighting the potential of AMA1 as a valuable component in serological tests (Kim et al., 2022).

Numerous studies have aimed to identify the ideal marker which can aid in distinguishing *T. gondii* acute or chronic infection, but there is a lack of investigation in OnSite tests. A study, utilizing 2D gel electrophoresis and IgA probing in human sera, identified the novel antigen—subtilisin-like protein (SUB1), which exhibited a higher likelihood of responding to specific IgA, IgM, and IgG in patients with acute rather than chronic *Toxoplasma* infection, as evidenced by a line blot test conducted on 80 human blood samples (Hruzik et al., 2011). In a separate study (Liu et al., 2012), screening toxoplasmosis human sera using 2D and LC/MS–MS revealed that 13 proteins recognized by IgG antibodies and 1 protein (ROP2) targeted IgM antibodies from group 1 (IgM negative/IgG positive) and group 2 (IgM positive/IgG negative), respectively. Afterward, by ELISA, the rROP2 fragment (186–533 aa) demonstrated specific capture of *Toxo*-IgM antibodies with 100% sensitivity (48/48) in group 2, while cross-reactivity was noted in 16% of individuals who were infected with *Leishmania* spp. and 10% of individuals infected with *P. vivax*. Recently, the well-established ELISA antigen GRA7 was assessed in an immunochromatographic test (ICT) involving 88 human sera samples. The ICT exhibited sensitivity ranging from 93.1 to 100% and specificity of 100% in detecting IgG and/or IgM antibodies, which was consistent with the performance observed in conventional ELISA methods (Ybanez and Nishikawa, 2020). Nevertheless, the GRA7-based ICT test did not independently examine IgM and IgG levels.

The immune system usually eliminates *T. gondii* tachyzoites rapidly, which results in prolonged encystment, a process favored in infections with low or moderately virulent strains in more resistant hosts (Lyons et al., 2002). As a result, cysts can maintain minimal immune system activation, allowing IgM antibodies to exist for an extended period. In contrast, the IgM response for the tachyzoite stage is short and diminishes rapidly. Thus, there may be some antigens that induce IgM during the tachyzoite stage but are quickly forgotten or induce IgM briefly. This circumstance could be one of the reasons why the sensitivity of one or several recombinant antigens does not fully match that of the tachyzoite whole lysate antigen in diagnostics.

In this study, utilizing the mouse sera collected from virulent tachyzoite RH strains, where the IgM response was robust but a short-lived, we successfully screened out three uncharacterized antigens of *T. gondii*, such as EF1γ, PGKI, and GAP50. Moreover, we established an on-site detection kit for IgM detection based on GAP50, demonstrating its effectiveness in both experimentally infected mice and human serum samples. It may raise concerns regarding the narrow detection window of several weeks, but it is important to emphasize that within this brief period, the robust IgM response provides a reliable indicator of recent *T. gondii* exposure. This plays a vital role in ensuring prompt treatment and illness prevention, especially for pregnant women. Although the IgM response generally declines quickly, there are reported cases of prolonged persistence.

The limitation of 2DE is that the presence of multiple proteins or multiple spots can correspond to a single protein caused by differential digestion or post-translational modifications. Therefore, three candidate antigens were examined for their ability to be used in diagnosis through *in silico* analysis and other complementary methods.

Based on a previous study (Tao et al., 2014), the elongation factor 1–gamma (EF1γ) was one of the 14 candidates who were highly expressed during *T. gondii* infection discovered by sera from infected pigs. In another publication, EF1γ also was found as a candidate biomarker for chronic periodontitis discovery via high-performance liquid chromatography and fragmentation using tandem MS of gingival crevicular fluid samples (Baliban et al., 2012). In addition, EF1γ is one of the four newly discovered immunogenic proteins of *P. multocida*, which is identified through 2-DE MALDI-TOF MS analysis with immune serum (Wang et al., 2021), or EF1γ mRNA overexpression was observed in biopsy specimens of esophageal carcinoma, suggesting its use as a possible indicator of tumor aggressiveness (Mimori et al., 1996). EF1γ was the most prominent intact protein identified by 2D Western blotting in our study, although this recombinant protein could not be detected by the IgM of *T. gondii*-infected mouse sera that need to be further investigated.

PGKI is a glycolytic enzyme targeted to the cytosol of *T. gondii* (Fleige et al., 2007), but there is very little information about this protein for diagnosis. Phosphoglycerate kinase initiates ATP generation in glycolysis. It is found in numerous parasites and has been identified as a promising target for vaccine and therapeutic development due to its distinct characteristics compared with human enzymes (Timson, 2016). In a previous study, antibodies raised against recombinant *C. sinensis* PGK demonstrated specificity toward native PGK from *C. sinensis* and effectively localized it within the muscular tissue and tegument of adult flukes. This suggests the potential utility of *C. sinensis* PGK as an immunoreagent for the serodiagnosis of clonorchiasis (Hong et al., 2000). Additionally, vaccination with *F. hepatica* phosphoglycerate kinase may prevent illness by inhibiting the energy production of fluke and disrupting the interaction between the surface-expressed enzyme and the host. However, the efficacy of protection varies significantly, ranging from 0 to 69%, depending on the delivery method and vaccine formulation (Jaros et al., 2010). During our investigation, PGKI and GAP50 were minor proteins identified by LC–MS/MS during 2DE Western blotting but were strongly detected by IgM as recombinant forms. Furthermore, our study revealed that PGKI-based ELISA faced problems with cross-reactivity with *P. vivax* and common human serum compared with GAP50.

The *T. gondii* GAP50 is a glycoprotein that functions as an integral membrane protein. It serves as an anchor for many components, including myosin A (TgMyoA), accompanying light chain (TgMLC1), actin, and TgGAP45. Together, these components form a glideosome, which plays a crucial role in facilitating motility necessary for host cell invasion (Fauquenoy et al., 2008; Harding et al., 2016). In other apicomplexan parasites such as *Plasmodium*, *Cryptosporidium*, and several species, the glideosome-associated protein GAP50 is also essential for cell penetration and substrate gliding motility via an actin–myosin motor (Bosch et al., 2012; Dearnley et al., 2012). Interestingly, the transmembrane protein PfGAP50 on developing gametes has been identified as a receptor for the host complement regulator factor H (FH). Plasmodium uses the surface-bound FH to inactivate the complement protein C3b, allowing it to avoid being eliminated by the complement system in the mosquito midgut. Interfering with FH-mediated protection, whether by neutralizing FH or inhibiting PfGAP50, significantly impairs gametogenesis and limits parasite transmission. Antibodies targeting PfGAP50 can prevent FH binding to the surface parasites, thereby destroying their ability to resist human complement. Consequently, PfGAP50 antibodies lead to reduced zygote numbers and lower infection rates of *Plasmodium* in mosquitoes (Dearnley et al., 2012).

However, unlike many major antigen candidates used in diagnosis, which are typically surface or secretory antigens, GAP50 was found to be localized inside the parasite plasma membrane. The database shows ortholog patterns in three other apicomplexan parasites, *P. falciparum*, *P. yoelii*, and *Eimeria tenella*, sharing 41–58% of identity across their entire sequences, except for the amino-terminal signal peptides (Gaskins et al., 2004). The high conservation of Tg-GAP50 orthologs in other apicomplexan parasites implies diagnostic assay cross-reactivity. Thus, it is imperative to assess GAP50 within the context of properly classified patient samples. In the future, there is potential to use GAP50 in combination with multiple rAgs to improve the accuracy and sensitivity of IgM detection in patient specimens.

In this study, GAP50 was analyzed for its potential application in rapid FICT. It is an efficient antigen for the detection of IgM antibodies in sera compared with the conventional ELISA based on *T. gondii* TLA. In contrast to the commercial OnSite kit, which utilizes rAgs for the detection of IgG and IgM separately, our system focused solely on IgM detection on a single strip. While the preliminary results are encouraging, further investigation is required to explore the potential incorporation of IgG detection within a strip. Although gold-based materials are durable and easily visible, it is important to note that relying on a visual assessment of the very faint strip may lead to subjective and varying interpretations, potentially causing inconsistent results. While fluorescence-based assays such as the FICT-GAP50 may necessitate specialized equipment such as fluorescence readers, they offer higher sensitivity, allowing for the detection of lower concentrations.

## 5 Conclusion

We have thoroughly characterized the *T. gondii* GAP50 protein, identifying it as a potential novel target for the specific detection of IgM antibodies in both rodent models and standard human patient sera when compared with EF1γ and PGKI. Subsequently, we successfully developed a rapid diagnostic fluorescent test capable of distinguishing IgM levels in *T. gondii*-infected samples from seronegative and cross-reactive samples. Notably, the FICT approach using GAP50 was more sensitive than TLA-based ELISA for detecting IgM levels in sera from rodents infected with *T. gondii*. The application of the novel GAP50 protein in the FICT holds promise for facilitating a rapid 20-min Point-of-Care Test (POCT) for the detection of *Toxoplasma gondii* IgM in clinical specimens in the future. However, it is essential to acknowledge that the lack of comprehensive assessment using properly classified patient sample in our study is one of the limitations of our research.

## Data availability statement

The mass spectrometry proteomics data have been deposited to the ProteomeXchange Consortium via the PRIDE partner repository with the dataset identifier PXD052664.

## Author contributions

M-NN: Data curation, Formal analysis, Methodology, Software, Validation, Visualization, Writing – original draft, Writing – review & editing. S-JY: Conceptualization, Data curation, Project administration, Supervision, Writing – review & editing. HP: Conceptualization, Funding acquisition, Investigation, Project administration, Resources, Supervision, Writing – review & editing.

## Ethics statement

Ethical approval was not required for the studies on humans in accordance with the local legislation and institutional requirements because only commercially available established cell lines were used. The animal study was approved by Animal Ethics Committee of Wonkwang University (WKU22-18). The study was conducted in accordance with the local legislation and institutional requirements.

## Footnotes

^1^ http://tools.iedb.org/main/tcell/

## Glossary

2DE: Two-dimensional gel electrophoresis
FICT: Fluorescence immunochromatographic test
EF1γ: Putative elongation factor 1-gamma
PGKI: Phosphoglycerate kinase 1
GAP50: Acid phosphatase GAP50
LOD: Limit of detection
AIDS: Acquired immune deficiency syndrome
Wpi: Weeks post-infection
Dpi: Days post-infection
POCT: Point-of-care test
TLA: Total lysate antigen
rAg: Recombinant antigen
SAG: Surface antigen
MAG: Matrix antigen
GRA: Dense granule antigen
ROP: Rhoptry antigen
MIC: Microneme antigen
MS analysis: Mass spectrometry analysis
*P. yoelii*: *Plasmodium yoelii*
*P. vivax*: *Plasmodium vivax*
LC/MS–MS: Liquid chromatography /tandem mass spectrometry
IPTG: Isopropyl-b-D-thiogalactopyranoside
Eu-NP: Europium nanoparticle
CL: Control line
TL: Test line
DW: Distilled water
SD: Standard deviation
i.p.: Intraperitoneal
BAG1: Bradyzoite antigens

## References

[ref1] Ajzenberg D. Yera H. Marty P. Paris L. Dalle F. Menotti J.. (2009). Genotype of 88 toxoplasma gondii isolates associated with toxoplasmosis in immunocompromised patients and correlation with clinical findings. J. Infect. Dis. 199, 1155–1167. doi: 10.1086/597477, PMID: 19265484

[ref2] Alvarado-Esquivel C. Niewiadomski A. Schweickert B. Liesenfeld O. (2011). Antiparasitic treatment suppresses production and avidity of toxoplasma gondii-specific antibodies in a murine model of acute infection*. Eur. J. Microbiol. Immunol. 1, 249–255. doi: 10.1556/EuJMI.1.2011.3.9, PMID: 24516731 PMC3906621

[ref3] Anand N. Lutshumba J. Whitlow M. Abdelaziz M. H. Mani R. Suzuki Y. (2022). Deficiency in indoleamine-2, 3-dioxygenase induces upregulation of guanylate binding protein 1 and inducible nitric oxide synthase expression in the brain during cerebral infection with toxoplasma gondii in genetically resistant BALB/c mice but not in genetically susceptible C57BL/6 mice. Microbes Infect. 24:104908. doi: 10.1016/j.micinf.2021.104908, PMID: 34781010 PMC9081123

[ref4] Anand N. Sehgal R. Kanwar R. K. Dubey M. L. Vasishta R. K. Kanwar J. R. (2015). Oral administration of encapsulated bovine lactoferrin protein nanocapsules against intracellular parasite toxoplasma gondii. Int. J. Nanomedicine 10, 6355–6369. doi: 10.2147/IJN.S85286, PMID: 26504384 PMC4605239

[ref5] Armbruster D. A. Pry T. (2008). Limit of blank, limit of detection and limit of quantitation. Clin. Biochem. Rev. 29, S49–S52, PMID: 18852857 PMC2556583

[ref6] Arranz-Solis D. Mukhopadhyay D. Saeij J. J. P. (2021). Toxoplasma effectors that affect pregnancy outcome. Trends Parasitol. 37, 283–295. doi: 10.1016/j.pt.2020.10.013, PMID: 33234405 PMC7954850

[ref7] Bachan M. Deb A. R. Maharana B. R. Sudhakar N. R. Sudan V. Saravanan B. C.. (2018). High seroprevalence of toxoplasma gondii in goats in Jharkhand state of India. Vet. Parasitol. Reg. Stud. Reports 12, 61–68. doi: 10.1016/j.vprsr.2018.02.004, PMID: 31014811

[ref8] Baliban R. C. Sakellari D. Li Z. DiMaggio P. A. Garcia B. A. Floudas C. A. (2012). Novel protein identification methods for biomarker discovery via a proteomic analysis of periodontally healthy and diseased gingival crevicular fluid samples. J. Clin. Periodontol. 39, 203–212. doi: 10.1111/j.1600-051X.2011.01805.x, PMID: 22092770 PMC3268946

[ref9] Begeman I. J. Lykins J. Zhou Y. Lai B. S. Levigne P. El Bissati K.. (2017). Point-of-care testing for toxoplasma gondii IgG/IgM using toxoplasma ICT IgG-IgM test with sera from the United States and implications for developing countries. PLoS Negl. Trop. Dis. 11:e0005670. doi: 10.1371/journal.pntd.0005670, PMID: 28650970 PMC5501679

[ref10] Bobic B. Sibalic D. Djurkovic-Djakovic O. (1991). High levels of IgM antibodies specific for toxoplasma gondii in pregnancy 12 years after primary toxoplasma infection. Case report. Gynecol. Obstet. Investig. 31, 182–184. doi: 10.1159/000293151, PMID: 2071060

[ref11] Bosch J. Paige M. H. Vaidya A. B. Bergman L. W. Hol W. G. (2012). Crystal structure of GAP50, the anchor of the invasion machinery in the inner membrane complex of plasmodium falciparum. J. Struct. Biol. 178, 61–73. doi: 10.1016/j.jsb.2012.02.009, PMID: 22387043 PMC3322287

[ref13] Centers for Disease Control and Prevention. (2023) Parasites – Toxoplasmosis (Toxoplasma infection). Available at: https://www.cdc.gov/parasites/toxoplasmosis/disease.html (Accessed June 2023)

[ref14] Chapey E. Wallon M. Peyron F. (2017). Evaluation of the LDBIO point of care test for the combined detection of toxoplasmic IgG and IgM. Clin. Chim. Acta 464, 200–201. doi: 10.1016/j.cca.2016.10.023, PMID: 27765564

[ref15] Dearnley M. K. Yeoman J. A. Hanssen E. Kenny S. Turnbull L. Whitchurch C. B.. (2012). Origin, composition, organization and function of the inner membrane complex of plasmodium falciparum gametocytes. J. Cell Sci. 125, 2053–2063. doi: 10.1242/jcs.099002 22328505

[ref16] Doskaya M. Liang L. Jain A. Can H. Gulce Iz S. Felgner P. L.. (2018). Discovery of new toxoplasma gondii antigenic proteins using a high throughput protein microarray approach screening sera of murine model infected orally with oocysts and tissue cysts. Parasit. Vectors 11:393. doi: 10.1186/s13071-018-2934-1, PMID: 29973272 PMC6033234

[ref17] Dubey J. (2010). Toxoplasmosis of animals and humans. second Edn. Boca Raton, FL: CRC Press LLC.

[ref18] Duong B. T. Than D. D. Ju B. G. Trinh T. T. Mok C. P. Jeong J. H.. (2022). Development of a rapid fluorescent diagnostic system for early detection of the highly pathogenic avian influenza H5 clade 2.3.4.4 viruses in chicken stool. Int. J. Mol. Sci. 23, 6301. doi: 10.3390/ijms23116301, PMID: 35682982 PMC9181406

[ref19] Fauquenoy S. Morelle W. Hovasse A. Bednarczyk A. Slomianny C. Schaeffer C.. (2008). Proteomics and glycomics analyses of N-glycosylated structures involved in toxoplasma gondii--host cell interactions. Mol. Cell. Proteomics 7, 891–910. doi: 10.1074/mcp.M700391-MCP200 18187410

[ref20] Ferra B. Holec-Gasior L. Gatkowska J. Dziadek B. Dzitko K. (2020). Toxoplasma gondii recombinant antigen AMA1: diagnostic utility of protein fragments for the detection of IgG and IgM antibodies. Pathogens 9, 43. doi: 10.3390/pathogens9010043 31948063 PMC7168680

[ref21] Flegr J. Prandota J. Sovickova M. Israili Z. H. (2014). Toxoplasmosis--a global threat. Correlation of latent toxoplasmosis with specific disease burden in a set of 88 countries. PLoS One 9:e90203. doi: 10.1371/journal.pone.0090203, PMID: 24662942 PMC3963851

[ref22] Fleige T. Fischer K. Ferguson D. J. Gross U. Bohne W. (2007). Carbohydrate metabolism in the toxoplasma gondii apicoplast: localization of three glycolytic isoenzymes, the single pyruvate dehydrogenase complex, and a plastid phosphate translocator. Eukaryot. Cell 6, 984–996. doi: 10.1128/EC.00061-07, PMID: 17449654 PMC1951530

[ref23] Gaskins E. Gilk S. DeVore N. Mann T. Ward G. Beckers C. (2004). Identification of the membrane receptor of a class XIV myosin in toxoplasma gondii. J. Cell Biol. 165, 383–393. doi: 10.1083/jcb.200311137, PMID: 15123738 PMC2172186

[ref24] Gatkowska J. M. Dziadek B. Dziadek J. Dzitko K. Dlugonska H. (2015). Recombinant MAG1 protein of toxoplasma gondii as a diagnostic antigen. Pol. J. Microbiol. 64, 55–59. doi: 10.33073/pjm-2015-007, PMID: 26094316

[ref25] Gomez C. A. Budvytyte L. N. Press C. Zhou L. McLeod R. Maldonado Y.. (2018). Evaluation of three point-of-care tests for detection of toxoplasma immunoglobulin IgG and IgM in the United States: proof of concept and challenges. Open Forum Infect. Dis. 5:ofy215. doi: 10.1093/ofid/ofy215, PMID: 30393749 PMC6204989

[ref26] Greenfield E. A. (2017). Sampling and preparation of mouse and rat serum. Cold Spring Harb. Protoc. 2017:pdb.prot100271. doi: 10.1101/pdb.prot100271 29093207

[ref27] Harding C. R. Egarter S. Gow M. Jimenez-Ruiz E. Ferguson D. J. Meissner M. (2016). Gliding associated proteins play essential roles during the formation of the inner membrane complex of toxoplasma gondii. PLoS Pathog. 12:e1005403. doi: 10.1371/journal.ppat.1005403, PMID: 26845335 PMC4742064

[ref28] Holec-Gasior L. (2013). Toxoplasma gondii recombinant antigens as tools for serodiagnosis of human toxoplasmosis: current status of studies. Clin. Vaccine Immunol. 20, 1343–1351. doi: 10.1128/CVI.00117-13, PMID: 23784855 PMC3889578

[ref29] Hong S. J. Seong K. Y. Sohn W. M. Song K. Y. (2000). Molecular cloning and immunological characterization of phosphoglycerate kinase from *Clonorchis sinensis*. Mol. Biochem. Parasitol. 108, 207–216. doi: 10.1016/s0166-6851(00)00220-6, PMID: 10838223

[ref30] Hruzik A. Asif A. R. Gross U. (2011). Identification of toxoplasma gondii SUB1 antigen as a marker for acute infection by use of an innovative evaluation method. J. Clin. Microbiol. 49, 2419–2425. doi: 10.1128/JCM.00464-11, PMID: 21543561 PMC3147871

[ref31] Jaros S. Jaros D. Wesolowska A. Zygner W. Wedrychowicz H. (2010). Blocking *Fasciola hepatica*'s energy metabolism – a pilot study of vaccine potential of a novel gene – phosphoglycerate kinase. Vet. Parasitol. 172, 229–237. doi: 10.1016/j.vetpar.2010.05.008 20538413

[ref32] Kang K. N. Choi I. U. Shin D. W. Lee Y. H. (2006). Cytokine and antibody responses of reactivated murine toxoplasmosis upon administration of dexamathasone. Korean J. Parasitol. 44, 209–219. doi: 10.3347/kjp.2006.44.3.209 16969058 PMC2532666

[ref33] Khan A. H. Noordin R. (2020). Serological and molecular rapid diagnostic tests for toxoplasma infection in humans and animals. Eur. J. Clin. Microbiol. Infect. Dis. 39, 19–30. doi: 10.1007/s10096-019-03680-2, PMID: 31428897 PMC7087738

[ref34] Kim M. J. Chu K. B. Mao J. Kang H. J. Eom G. D. Yoon K. W.. (2022). Recombinant AMA1 virus-like particle antigen for Serodiagnosis of toxoplasma gondii infection. Biomedicines 10, 2812. doi: 10.3390/biomedicines10112812, PMID: 36359332 PMC9687185

[ref35] Liesenfeld O. Press C. Montoya J. G. Gill R. Isaac-Renton J. L. Hedman K.. (1997). False-positive results in immunoglobulin M (IgM) toxoplasma antibody tests and importance of confirmatory testing: the Platelia Toxo IgM test. J. Clin. Microbiol. 35, 174–178. doi: 10.1128/jcm.35.1.174-178.1997, PMID: 8968902 PMC229533

[ref36] Liu L. Liu T. Yu L. Cai Y. Zhang A. Xu X.. (2012). rROP2(186-533): a novel peptide antigen for detection of IgM antibodies against toxoplasma gondii. Foodborne Pathog. Dis. 9, 7–12. doi: 10.1089/fpd.2011.0942, PMID: 22085219 PMC3250629

[ref37] Lourido S. (2019). Toxoplasma gondii. Trends Parasitol. 35, 944–945. doi: 10.1016/j.pt.2019.07.001 31345768

[ref38] Luo J. Wan J. Tang Z. Shen S. (2019). Identification of novel antigens for serum IgG diagnosis of human toxoplasmosis. Exp. Parasitol. 204:107722. doi: 10.1016/j.exppara.2019.107722, PMID: 31279928

[ref39] Lutshumba J. Ochiai E. Sa Q. Anand N. Suzuki Y. (2020). Selective upregulation of transcripts for six molecules related to T cell Costimulation and phagocyte recruitment and activation among 734 immunity-related genes in the brain during perforin-dependent, CD8(+) T cell-mediated elimination of toxoplasma gondii cysts. mSystems 5, e00189–20. doi: 10.1128/mSystems.00189-20 32291349 PMC7159899

[ref40] Lyons R. E. McLeod R. Roberts C. W. (2002). Toxoplasma gondii tachyzoite-bradyzoite interconversion. Trends Parasitol. 18, 198–201. doi: 10.1016/s1471-4922(02)02248-1, PMID: 11983592

[ref41] Mimori K. Mori M. Inoue H. Ueo H. Mafune K. Akiyoshi T.. (1996). Elongation factor 1 gamma mRNA expression in oesophageal carcinoma. Gut 38, 66–70. doi: 10.1136/gut.38.1.66, PMID: 8566862 PMC1382981

[ref42] Montoya J. G. Liesenfeld O. (2004). Toxoplasmosis. Lancet 363, 1965–1976. doi: 10.1016/S0140-6736(04)16412-X 15194258

[ref43] Nayeri T. Sarvi S. Moosazadeh M. Amouei A. Hosseininejad Z. Daryani A. (2020). The global seroprevalence of anti-toxoplasma gondii antibodies in women who had spontaneous abortion: a systematic review and meta-analysis. PLoS Negl. Trop. Dis. 14:e0008103. doi: 10.1371/journal.pntd.0008103, PMID: 32168351 PMC7069604

[ref44] Nguyen N. M. Duong B. T. Azam M. Phuong T. T. Park H. Thuy P. T. B.. (2019). Diagnostic performance of dengue virus envelope domain III in acute dengue infection. Int. J. Mol. Sci. 20, 3464. doi: 10.3390/ijms20143464, PMID: 31311082 PMC6679088

[ref45] NIBSC. (2020) Instructions for use (version 6.0, dated 16/01/2020)). WHO international standard 4th IS for antibodies, human, to toxoplasma gondii NIBSC code: 13/132.

[ref46] Oliveira C. B. Meurer Y. S. Andrade J. M. Costa M. E. Andrade M. M. Silva L. A.. (2016). Pathogenicity and phenotypic sulfadiazine resistance of toxoplasma gondii isolates obtained from livestock in northeastern Brazil. Mem. Inst. Oswaldo Cruz 111, 391–398. doi: 10.1590/0074-02760150459, PMID: 27276184 PMC4909038

[ref47] Poshtehban A. Shojaee S. Keshavarz H. Salimi M. Mohebali M. (2017). Comparison of the toxoplasma gondii mice and cell culture derived antigens in ELISA assay. Trop. Biomed. 34, 433–436, PMID: 33593025

[ref48] Rahimi-Esboei B. Zarei M. Mohebali M. Valian H. K. Shojaee S. Mahmoudzadeh R.. (2018). Serologic tests of IgG and IgM antibodies and IgG avidity for diagnosis of ocular toxoplasmosis. Korean J. Parasitol. 56, 147–152. doi: 10.3347/kjp.2018.56.2.147, PMID: 29742869 PMC5976017

[ref49] Reis M. M. Tessaro M. M. D'Azevedo P. A. (2006). Toxoplasma-IgM and IgG-avidity in single samples from areas with a high infection rate can determine the risk of mother-to-child transmission. Rev. Inst. Med. Trop. São Paulo 48, 93–98. doi: 10.1590/s0036-46652006000200007, PMID: 16699631

[ref50] Rijpkema S. Hockley J. Rigsby P. Guy E. C. Toxoplasma Study, G (2016). Establishment of replacement international standard 13/132 for human antibodies to toxoplasma gondii. Biologicals 44, 448–455. doi: 10.1016/j.biologicals.2016.04.006, PMID: 27378430

[ref51] Santoro F. Afchain D. Pierce R. Cesbron J. Y. Ovlaque G. Capron A. (1985). Serodiagnosis of toxoplasma infection using a purified parasite protein (P30). Clin. Exp. Immunol. 62, 262–269, PMID: 2417762 PMC1577441

[ref52] Silva D. A. Silva N. M. Mineo T. W. Pajuaba Neto A. A. Ferro E. A. Mineo J. R. (2002). Heterologous antibodies to evaluate the kinetics of the humoral immune response in dogs experimentally infected with toxoplasma gondii RH strain. Vet. Parasitol. 107, 181–195. doi: 10.1016/s0304-4017(02)00132-2, PMID: 12127249

[ref53] Simon L. Fillaux J. Guigon A. Lavergne R. A. Villard O. Villena I.. (2020). Serological diagnosis of toxoplasma gondii: analysis of false-positive IgG results and implications. Parasite 27:7. doi: 10.1051/parasite/2020006, PMID: 32031519 PMC7006501

[ref54] Tao Q. Xiao J. Wang Y. Fang K. Li N. Hu M.. (2014). Identification of genes expressed during toxoplasma gondii infection by in vivo-induced antigen technology (IVIAT) with positive porcine sera. J. Parasitol. 100, 470–479. doi: 10.1645/13-240.1 24646180

[ref55] Teimouri A. Modarressi M. H. Shojaee S. Mohebali M. Rezaian M. Keshavarz H. (2019). Development, optimization, and validation of an in-house dot-ELISA rapid test based on SAG1 and GRA7 proteins for serological detection of toxoplasma gondii infections. Infect Drug Resist 12, 2657–2669. doi: 10.2147/IDR.S219281, PMID: 31695442 PMC6717716

[ref56] Teimouri A. Mohtasebi S. Kazemirad E. Keshavarz H. (2020). Role of toxoplasma gondii IgG avidity testing in discriminating between acute and chronic toxoplasmosis in pregnancy. J. Clin. Microbiol. 58, e00505–20. doi: 10.1128/JCM.00505-20, PMID: 32321784 PMC7448626

[ref57] Timson D. J. (2016). Metabolic enzymes of helminth parasites: potential as drug targets. Curr. Protein Pept. Sci. 17, 280–295. doi: 10.2174/1389203717999160226180733 26983888

[ref58] Trees A. J. Crozier S. J. Buxton D. Blewett D. A. (1989). Serodiagnosis of ovine toxoplasmosis: an assessment of the latex agglutination test and the value of IgM specific titres after experimental oocyst-induced infections. Res. Vet. Sci. 46, 67–72. doi: 10.1016/S0034-5288(18)31120-2, PMID: 2646662

[ref59] Turunen H. Vuorio K. A. Leinikki P. O. (1983). Determination of IgG, IgM and IgA antibody responses in human toxoplasmosis by enzyme-linked immunosorbent assay (ELISA). Scand. J. Infect. Dis. 15, 307–311. doi: 10.3109/inf.1983.15.issue-3.12, PMID: 6648374

[ref60] Vargas-Villavicencio J. A. Canedo-Solares I. Correa D. (2022). Anti-toxoplasma gondii IgM long persistence: what are the underlying mechanisms? Microorganisms 10, 1659. doi: 10.3390/microorganisms10081659, PMID: 36014077 PMC9415799

[ref61] Wang F. Wang X. Ai W. Zeng D. Liang W. Hua L.. (2021). Three novel immunogenic proteins determined through 2-dimensional electrophoresis and mass spectrometry with immune serum confer protection against challenge with porcine *Pasteurella multocida* in mouse models. Res. Vet. Sci. 136, 303–309. doi: 10.1016/j.rvsc.2021.03.013, PMID: 33744821

[ref62] Ybanez R. H. D. Kyan H. Nishikawa Y. (2020). Detection of antibodies against toxoplasma gondii in cats using an immunochromatographic test based on GRA7 antigen. J. Vet. Med. Sci. 82, 441–445. doi: 10.1292/jvms.19-0654, PMID: 32037381 PMC7192716

[ref63] Ybanez R. H. D. Nishikawa Y. (2020). Serological detection of T. Gondii infection in humans using an immunochromatographic assay based on dense granule protein 7. Parasitol. Int. 76:102089. doi: 10.1016/j.parint.2020.102089, PMID: 32092466

[ref64] Ybanez R. H. D. Ybanez A. P. Nishikawa Y. (2020). Review on the current trends of toxoplasmosis Serodiagnosis in humans. Front. Cell. Infect. Microbiol. 10:204. doi: 10.3389/fcimb.2020.00204, PMID: 32457848 PMC7227408

