# Supplementary material for: Identification of novel biomarkers for anti-Toxoplasma gondii IgM detection and the potential application in rapid diagnostic fluorescent tests
Source: Front Microbiol. 2024 Jun 4;15:1385582. doi: 10.3389/fmicb.2024.1385582 (PMC11184589; doi:10.3389/fmicb.2024.1385582)
Supplement: Supplementary file 1 [file Data_Sheet_1.docx]

**Identification of novel biomarkers for anti-*Toxoplasma gondii* IgM detection and the potential application in rapid diagnostic fluorescent tests**

Minh-Ngoc Nguyen^1^, Seon-Ju Yeo^2.3*^ and Hyun Park^1^*

^1^Zoonosis Research Center, Department of Infection Biology, School of Medicine, Wonkwang University, Iksan 54538, Republic of Korea.

^2^Department of Tropical Medicine and Parasitology, Department of Biomedical Sciences, College of Medicine, Seoul National University, Seoul, 03080, Republic of Korea.

^3^Department of Tropical Medicine and Parasitology, Medical Research Center, Institute of Endemic Diseases, Seoul National University, Seoul, 03080, Republic of Korea.

*Correspondence: [hyunpk@wku.ac.kr](mailto:hyunpk@wku.ac.kr) (H.P.) and [yeosj@snu.ac.kr](mailto:yeosj@snu.ac.kr) (S.-J.Y.)

**Supplementary Information**


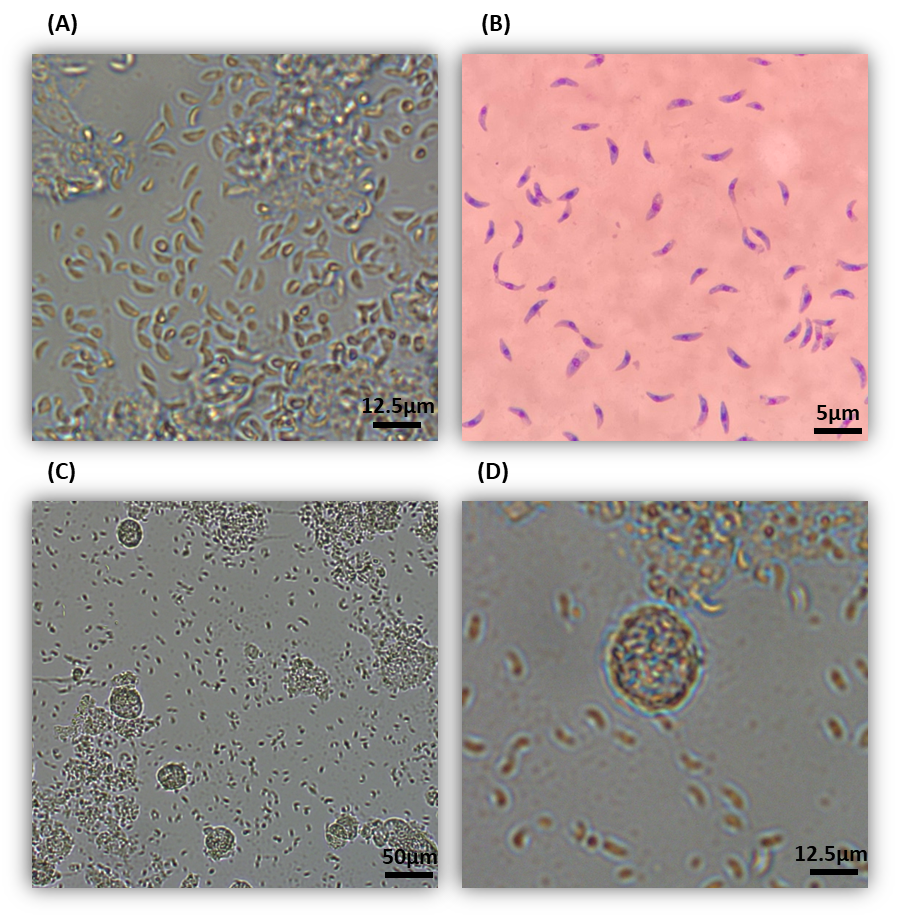


**FIGURE 9 -S1. In vitro cultured Toxoplasma gondii RH strain tachyzoites.**

(A) Tachyzoite-infected cells *in vitro* (magnification ×40). (B) Pure *T. gondii* tachzyoites (magnification ×100).


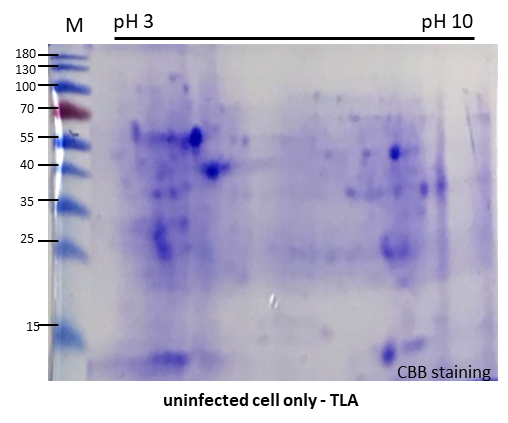


**FIGURE 10-S2. Two-dimension electrophoresis (2DE) of negative control-uninfected cells**


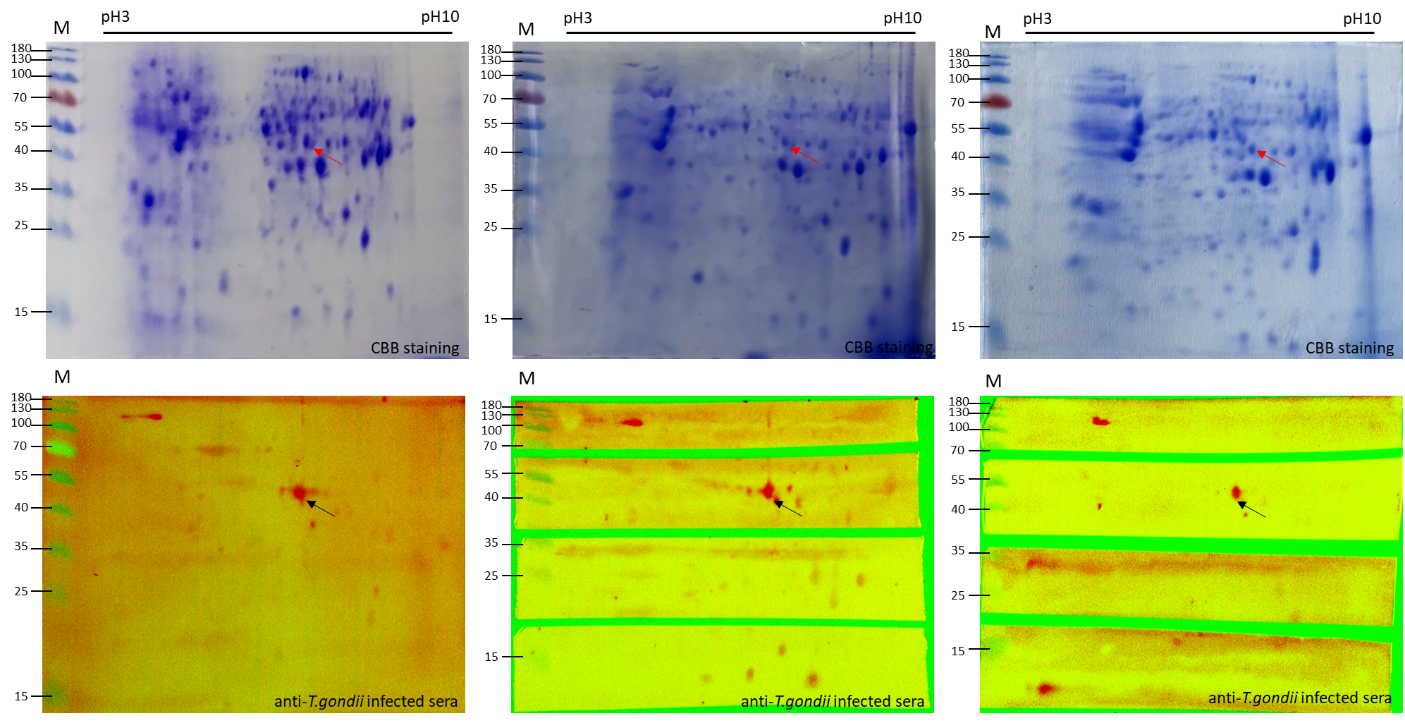


**FIGURE 11-S3. Identification of IgM-specific spot by 2D-western blotting with *T. gondii*-infected mouse sera.**

Triplicate two-dimensional electrophoresis (2DE) for proteomic analysis of *Toxoplasma gondii* tachyzoites was performed using an IPG strip at pH 3-10, then all proteins were transferred onto a PVDF membrane. After that, 10^6^ *T. gondii*-infected mouse sera – 5dpi were probed in comparison with normal sera diluted 1:100, followed by the detection of anti-mouse IgM-HRP antibodies.


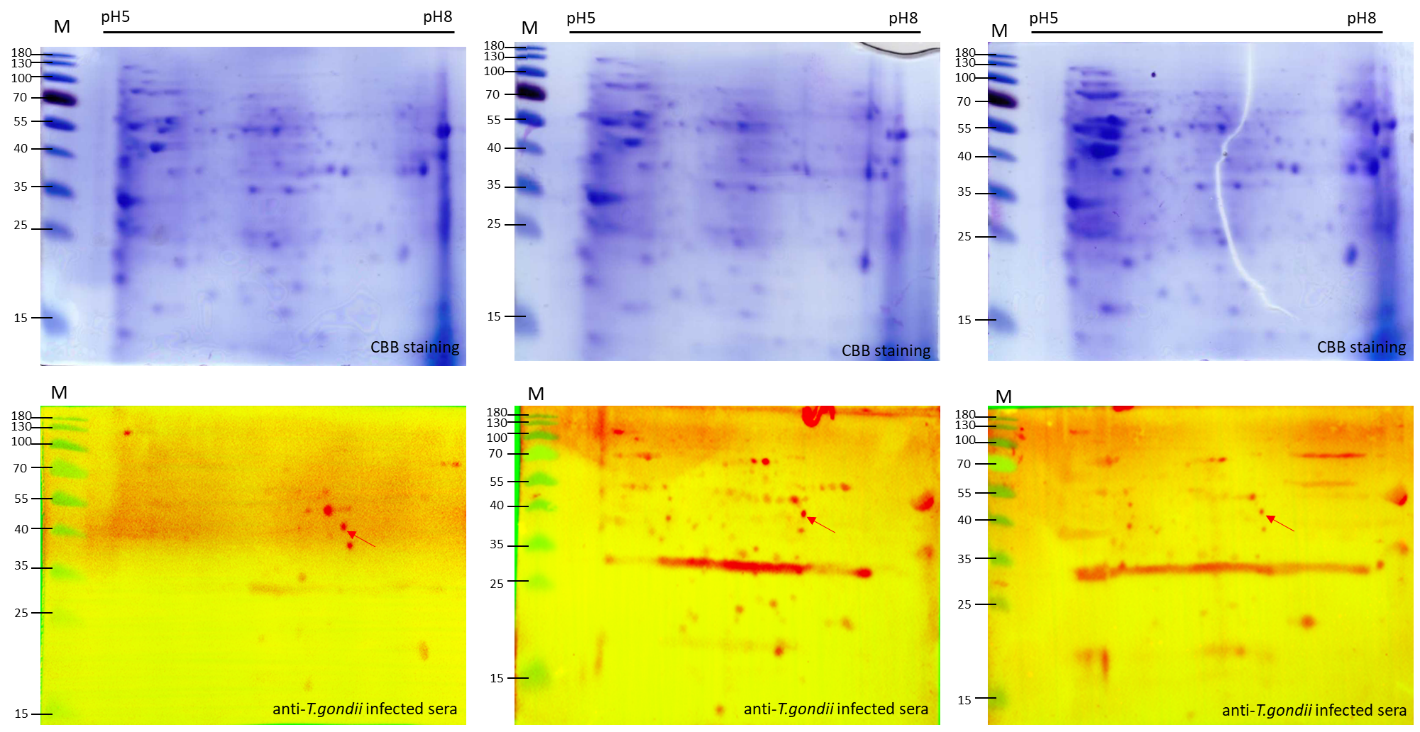


**FIGURE 12-S4. Identification of IgM-specific spot by 2D-western blotting with *T. gondii*-infected mouse.**

Triplicate two-dimensional electrophoresis (2DE) for proteomic analysis of *Toxoplasma gondii* tachyzoites was performed using an IPG strip at pH 5-8, then all proteins were transferred onto a PVDF membrane. After that, 10^6^ *T. gondii*-infected mouse sera – 5dpi were probed in comparison with normal sera diluted 1:100, followed by the detection of anti-mouse IgM-HRP antibodies.


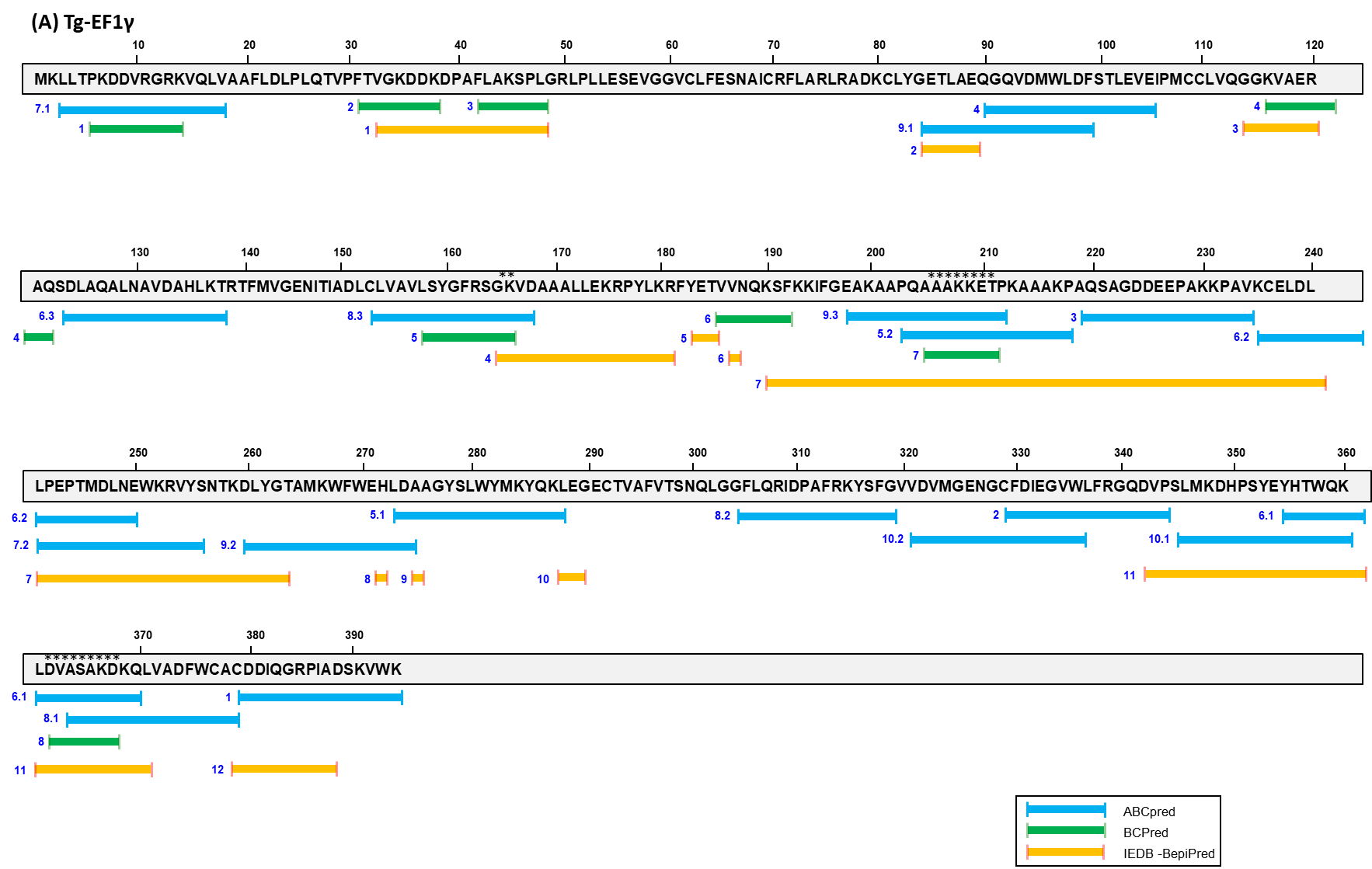


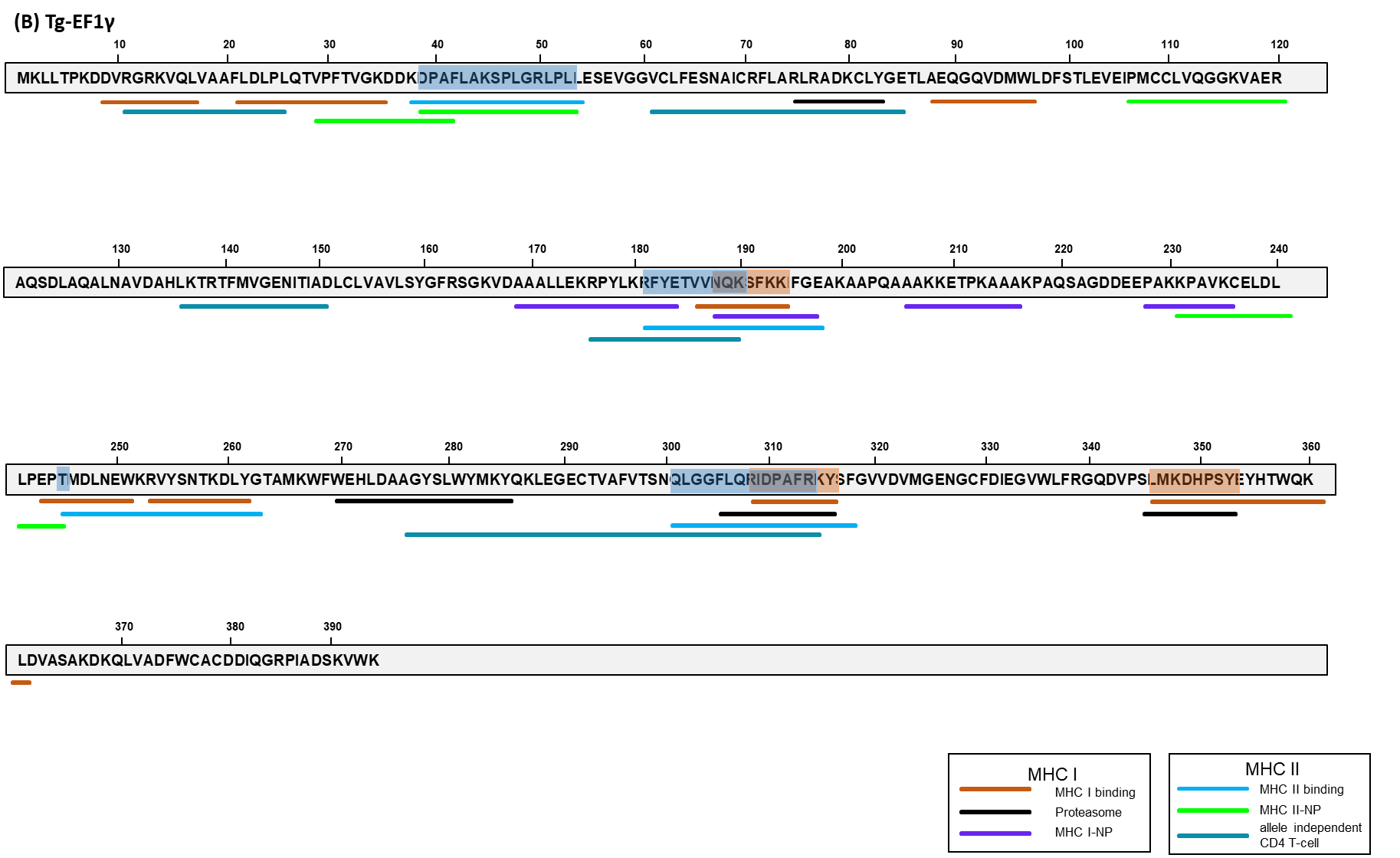


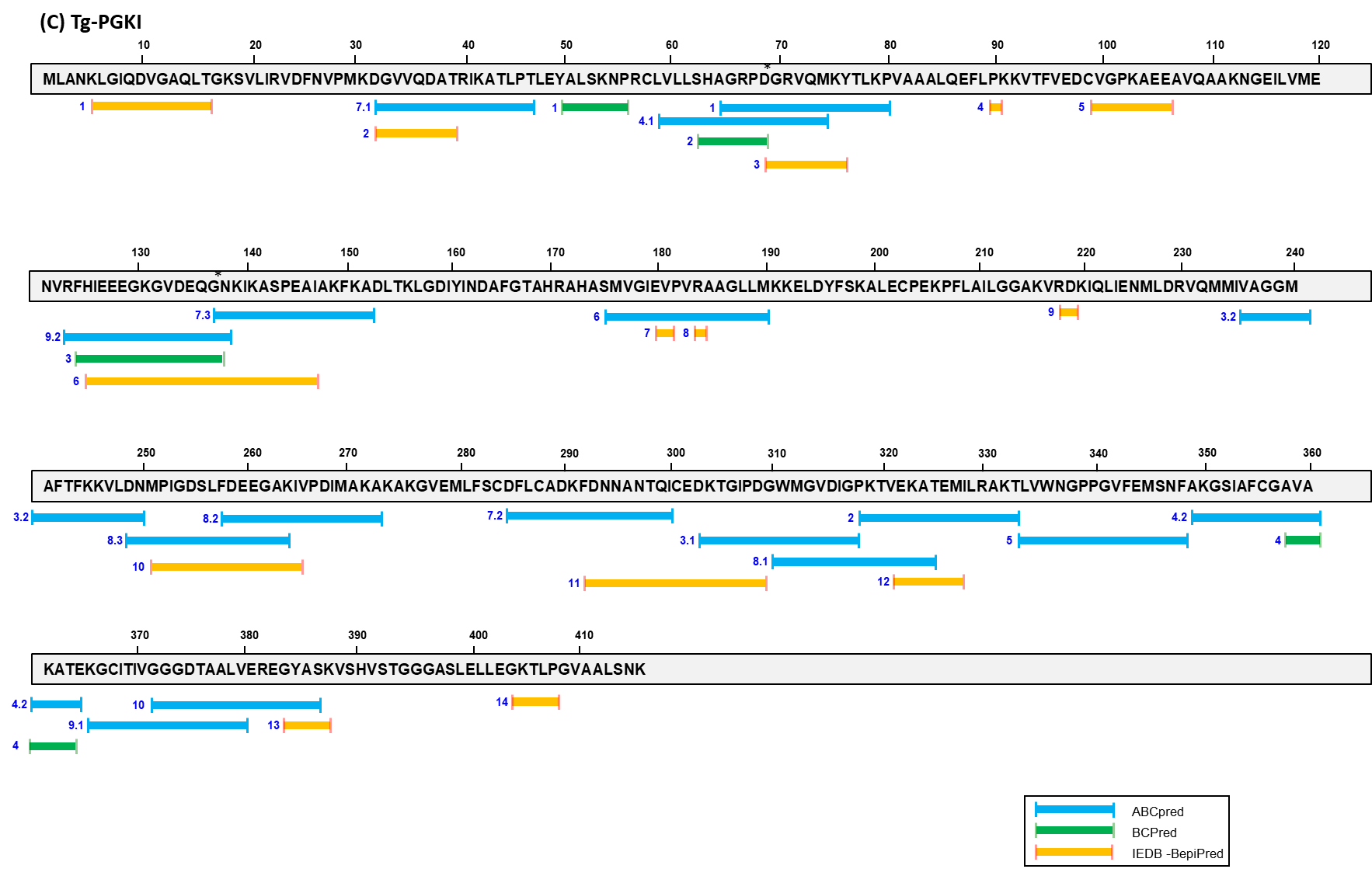


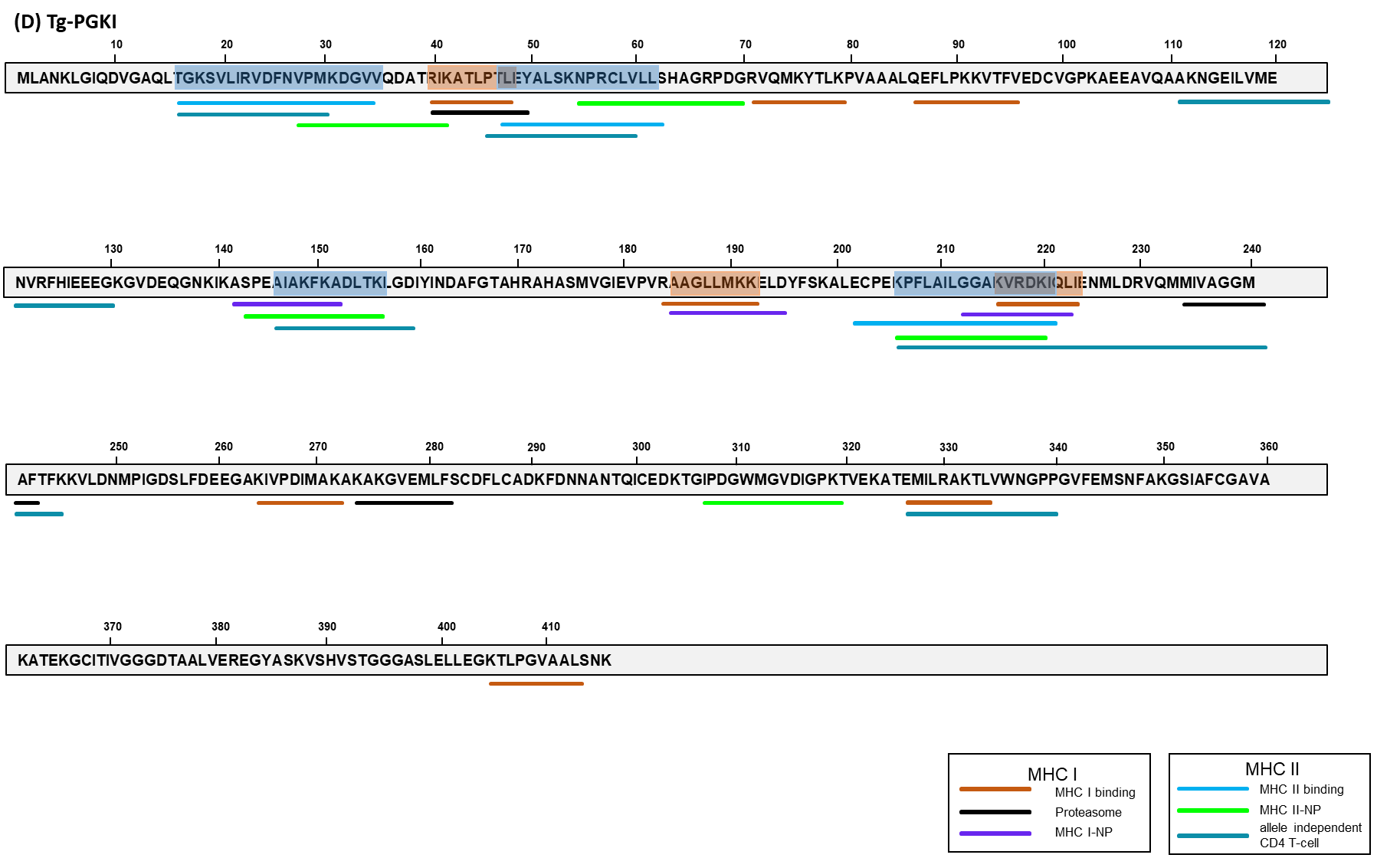


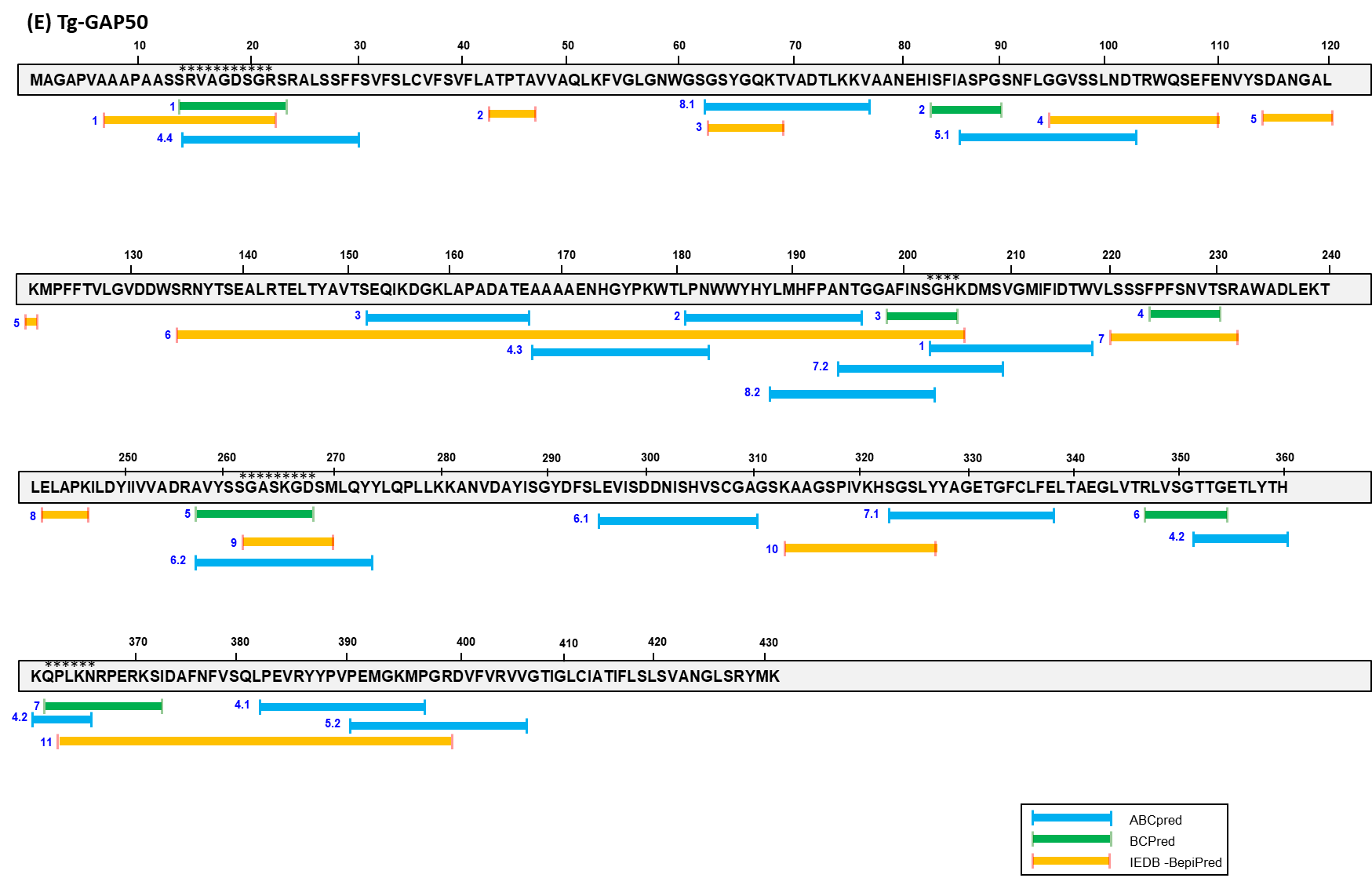


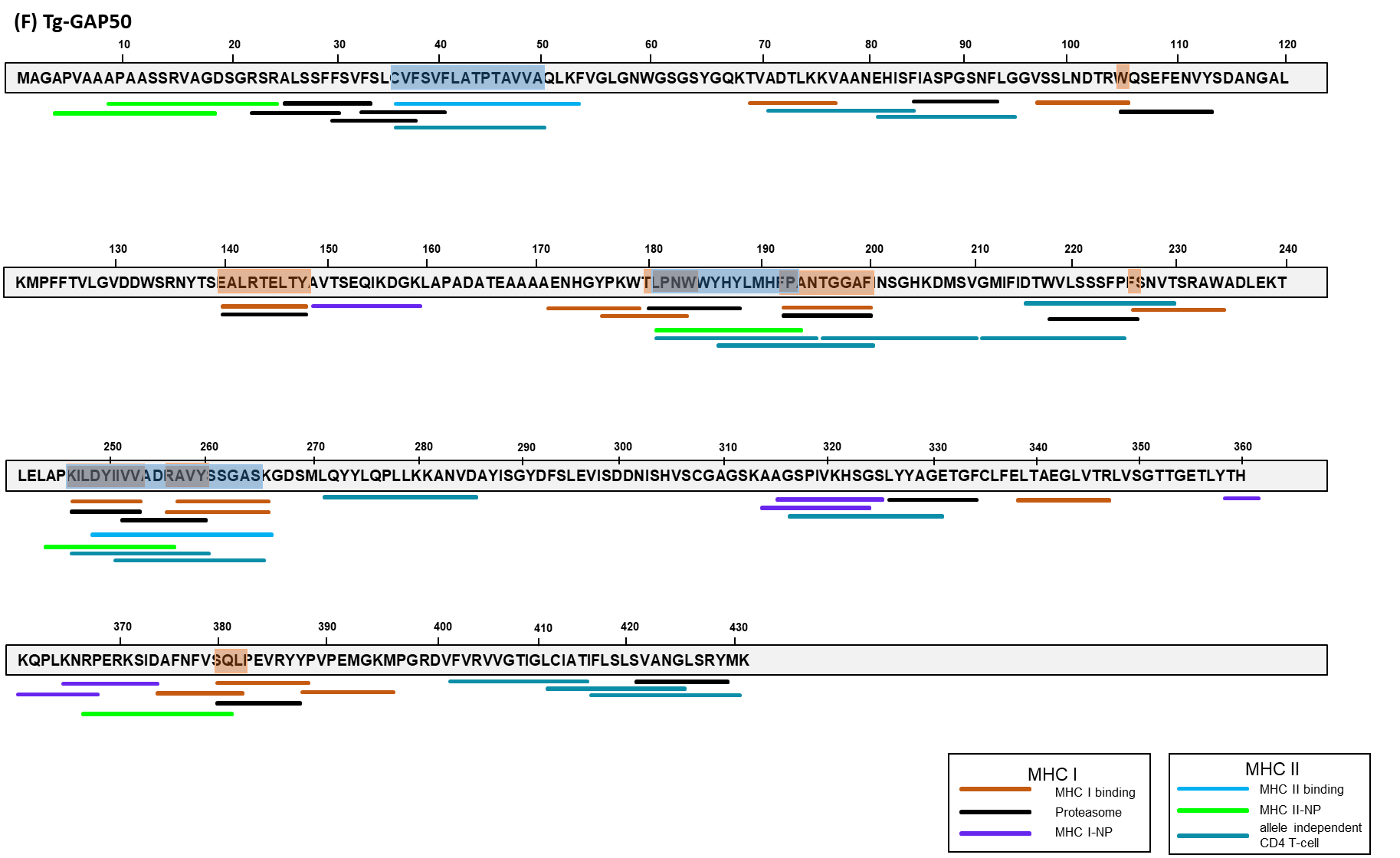


**FIGURE 13-S5: In silicon analysis of the novel biomarkers.**

Each amino acid sequence of (A, B) EF1γ, (C, D) PGKI, and (E, F) GAP50 was analyzed for potential linear epitope by three independent programs (ABCpred, BCPreds, and immune epitope database and analysis resource [IEDB]-BepiPred) (A, C, E). The peptides expected to be subjected to T cell epitope processing and bind with MHC class I and II molecules were analyzed through the EIDB Analysis resource tool (B, D, F).

Dots indicate common epitope sequences found in the three programs (Table 2). Brown and blue rectangles indicate the common MHC I-and MHC II-binding peptides found by the 2/3 programs, respectively.


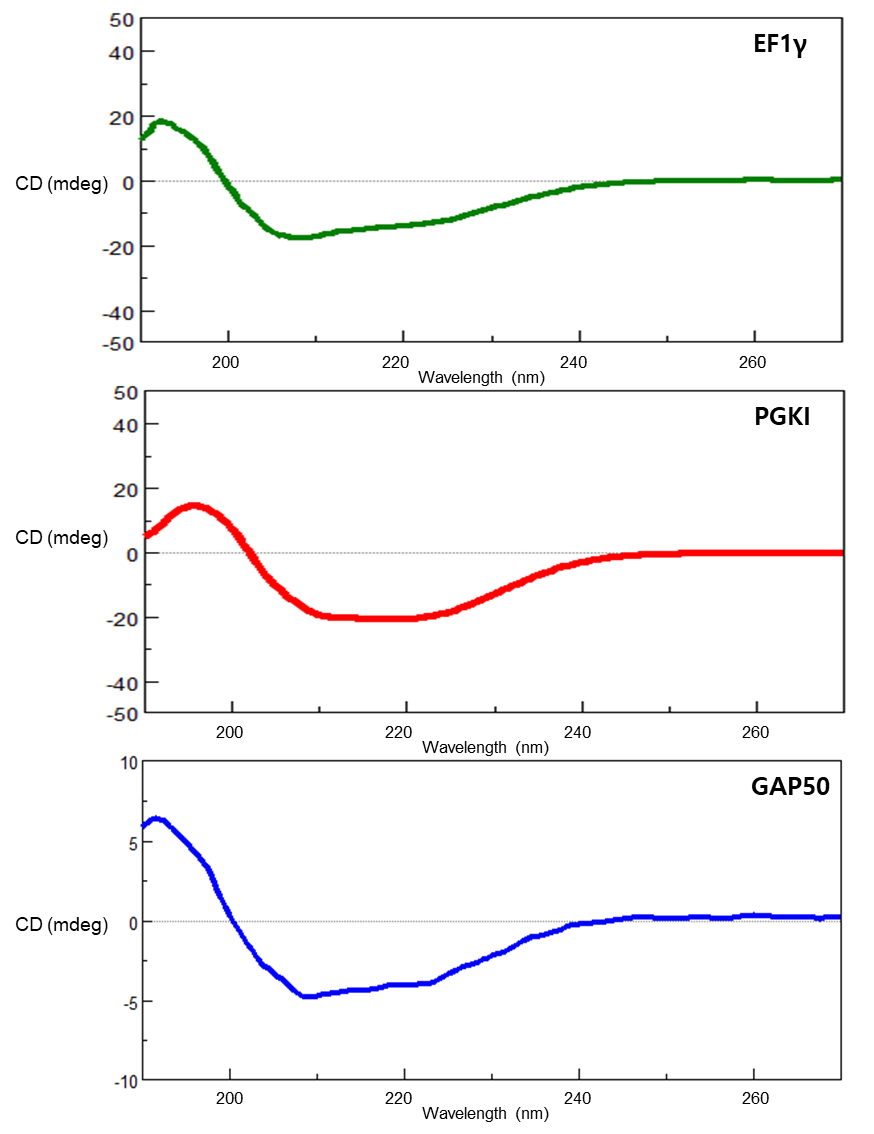


**FIGURE 14-S6. CD spectra of proteins with representative secondary structures**

EF1γ, PGI, GAP 50 recombinant protein in PBS were subjected to measurement CD with 0.2-cm pathlength cell.


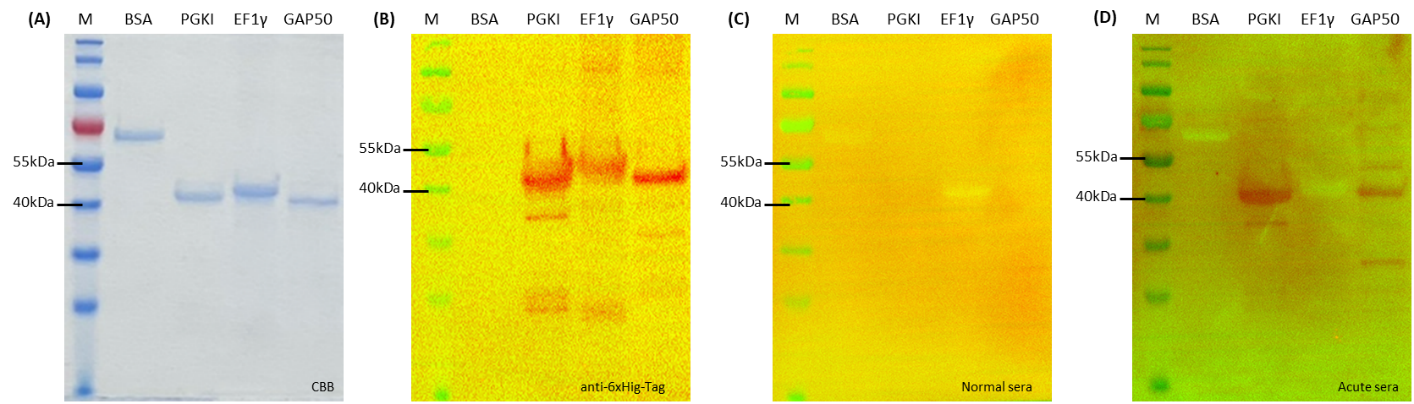


**FIGURE 15-S7. Immunoblotting of the recombinant antigens.**

(A) SDS-PAGE CBB staining BSA as the negative antigen in comparison with recombinant antigen *T. gondii*-EF1γ purified - target 46 kDa; PGKI purified - target 48.5 kDa, and GAP50 purified -target 50 kDa. (B) Western blotting using anti-6× Hig-Tag mouse IgG to confirm recombinant antigen expression and purification of *T. gondii*-EF1γ purified - target 46 kDa; PGKI purified - target 48.5 kDa, and GAP50 purified - target 50 kDa. (C; D) Western blotting to detect IgM antibodies probed by (C) normal mouse sera and (D) *T. gondii* (10^6^) tachyzoite-infected mouse sera as primary antibody of BSA in comparison with recombinant antigens *T. gondii*-EF1γ, PGKI, and GAP50.

Protein was run with 20 µg/lane. Anti-6×Hig-Tag mouse IgG-HRP was diluted 1:10000 in 5% non-fat milk. Normal and *T.gondii* infected mouse sera were diluted 1:100 in 5% BSA. A total of 10^6^ *T. gondii* tachzyoites of RH strain-infected mouse sera collected at 5 dpi were used. Goat anti-mouse IgM (heavy chain)-HRP was diluted 1:3000 in 5% BSA. M, Marker PageRuler Prestained Protein Ladder (#26617-Thermo Scientific).


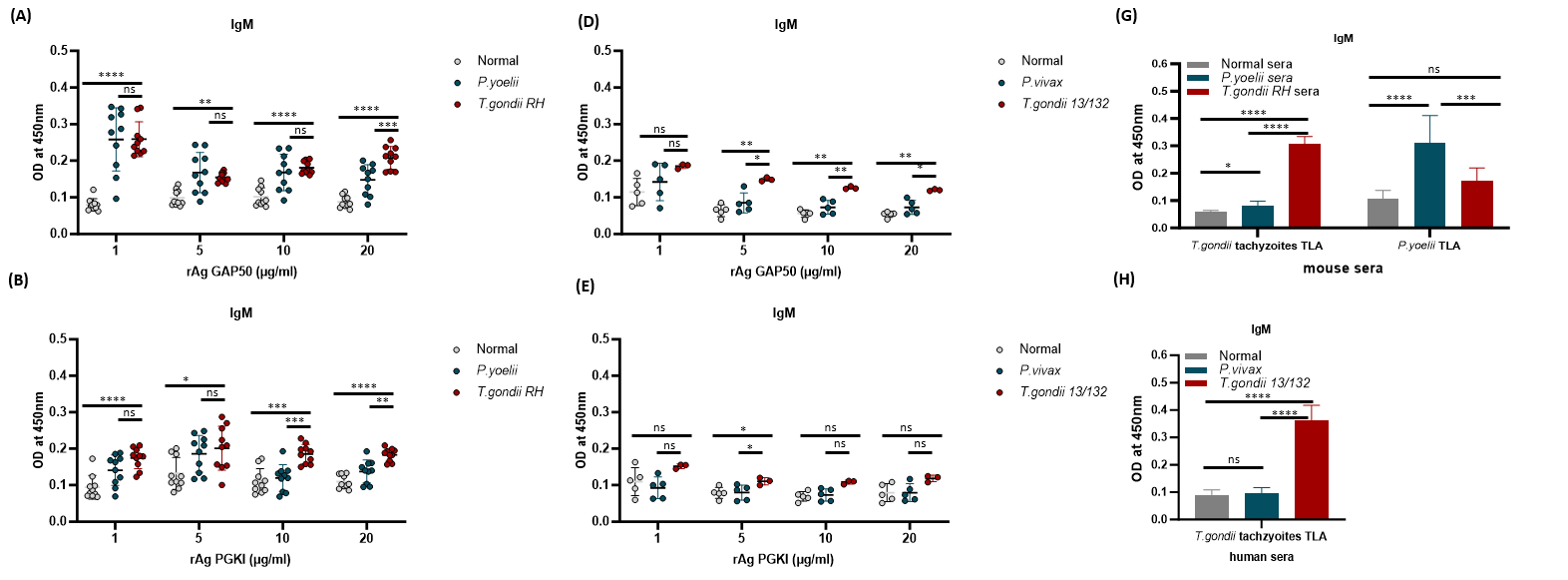


**FIGURE 16-S8. ELISA of rAg GAP50 and PGKI.**

Determination of IgM antibodies in mouse sera *T. gondii*-infected 10^6^ tachyzoites, 5dpi (A, B) and standard human sera infected *T.gondii* 13/132 (D, E) using recombinant GAP50 (A, D) and PGKI (B, E) in comparison with tachyzoite total lysate antigen (TLA) coated plates (G, H).

The recombinant *T. gondii*-PGKI and GAP50 were used with 1, 5, 10, and 20 µg/well. Total lysate antigen of tachyzoites and *P. yoelii* was used with 20 and 1 µg/well, respectively.

The ELISA data (each mouse group *n* =10; each human group *n*=5, standard human sera *T.gondii* 13/132 *n* =3) was shown as means ± SD.

ns: not statistically significant, **P* < 0.05, ***P* < 0.01, ****P* < 0.001, *****P* < 0.0001.


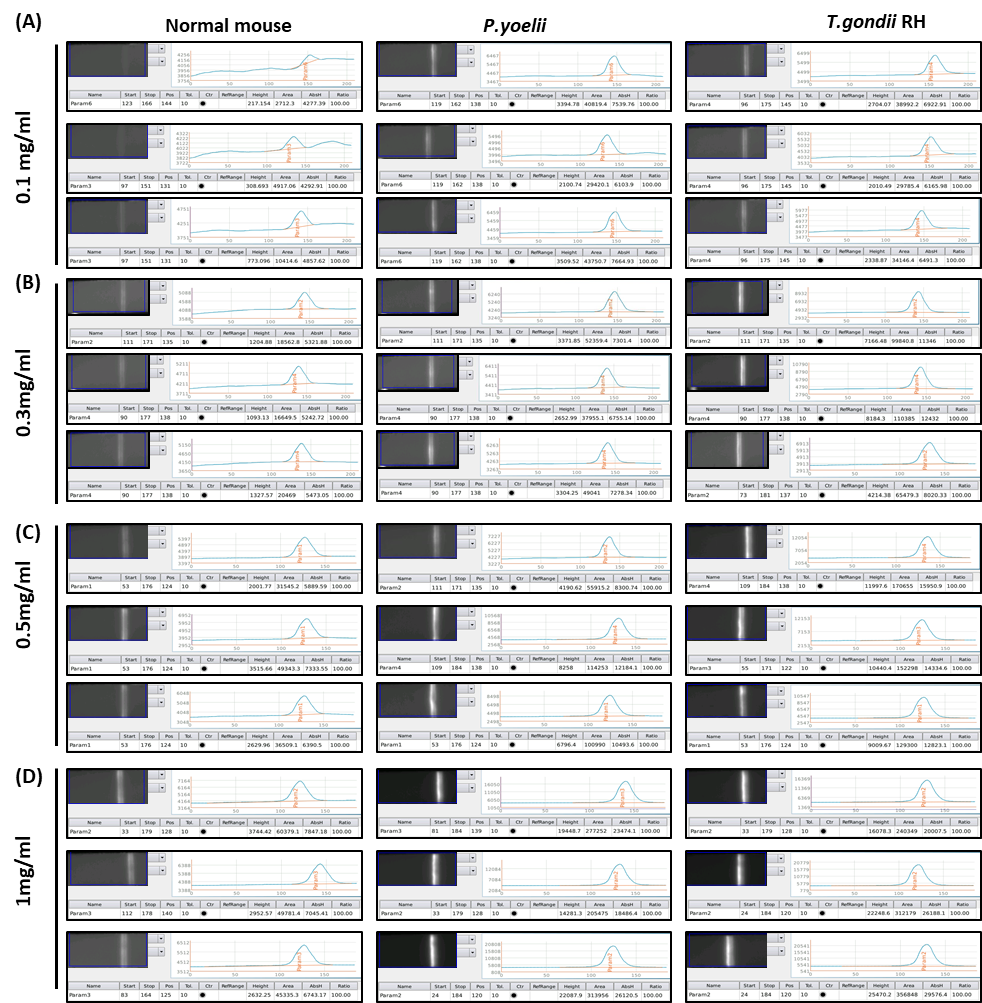


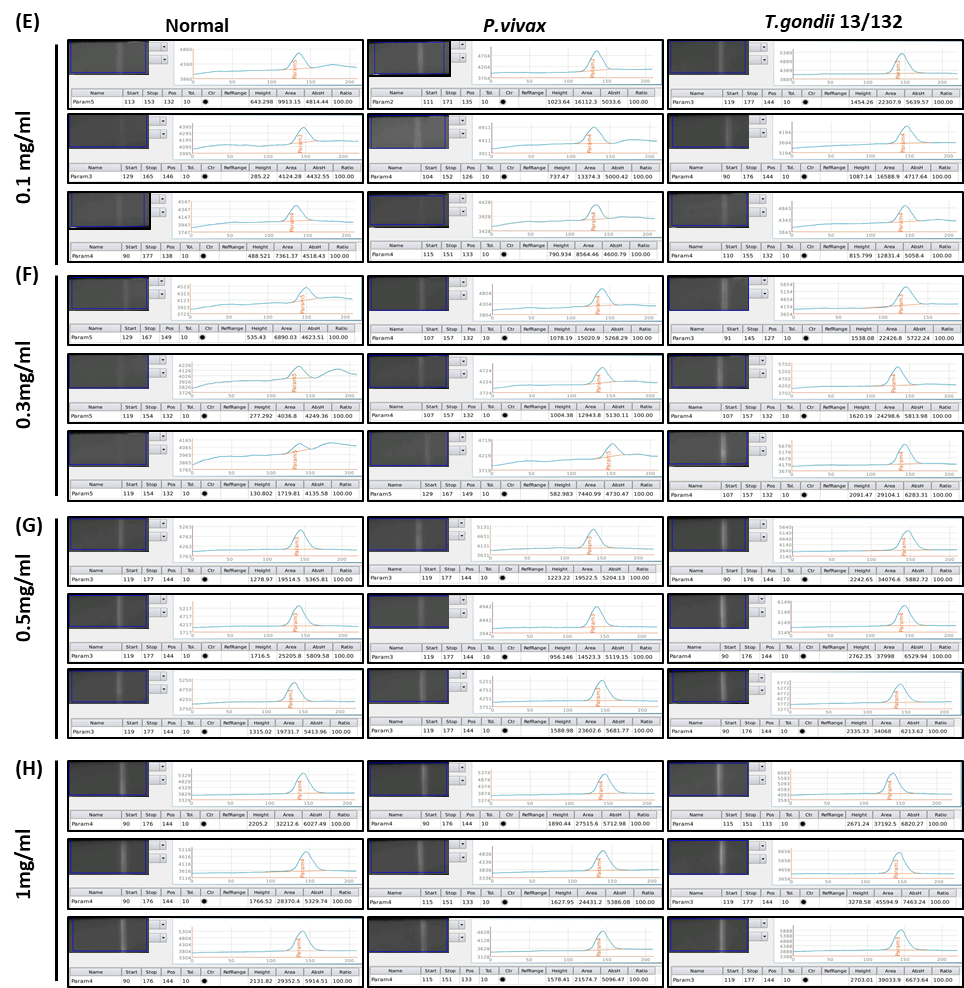


**FIGURE 17-S9. Raw data for Figure 5. Optimization of the rAg concentration coating strip for FICT.**

The NC membrane was coated with (A, E) 0.1, (B, F) 0.3, (C, G) 0.5, and (D, H) 1 mg/mL purified rAg *T.g*-GAP50. Mouse sera (A, B, C, and D) and/or (E, F, G, and H) were detected using an immunochromatographic test strip with Eu NP-conjugated anti-mouse/human IgM. The interaction of rAg with Abs present in sera was determined by measuring the fluorescence intensity (365 nm excitation and 610 nm emission). Two microliters of serum were used per reaction.


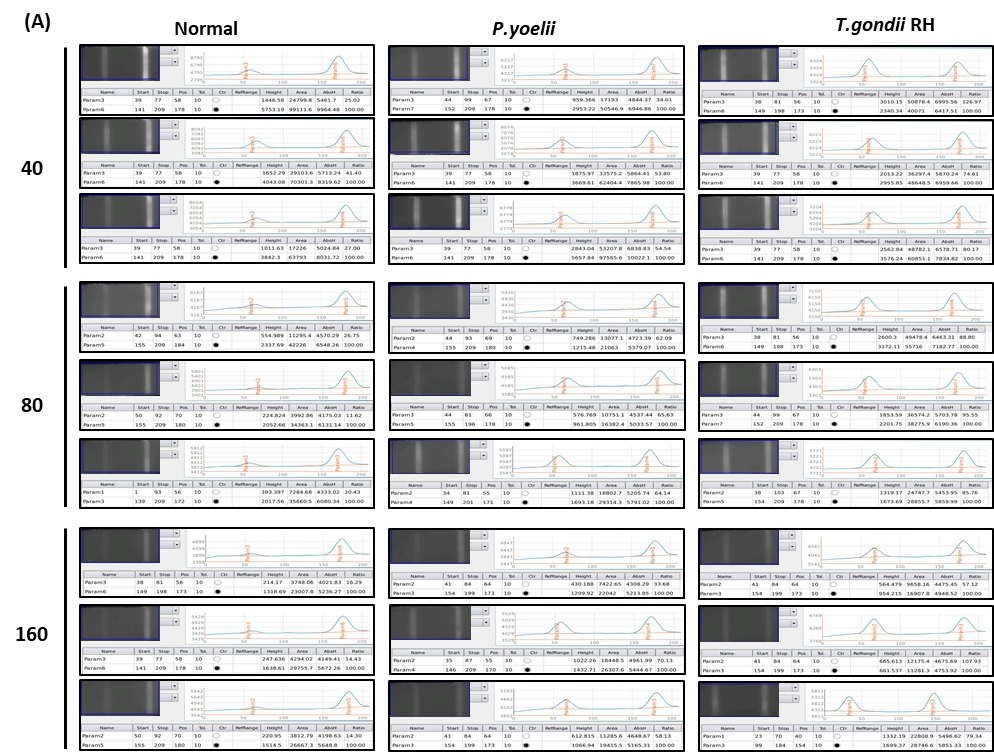


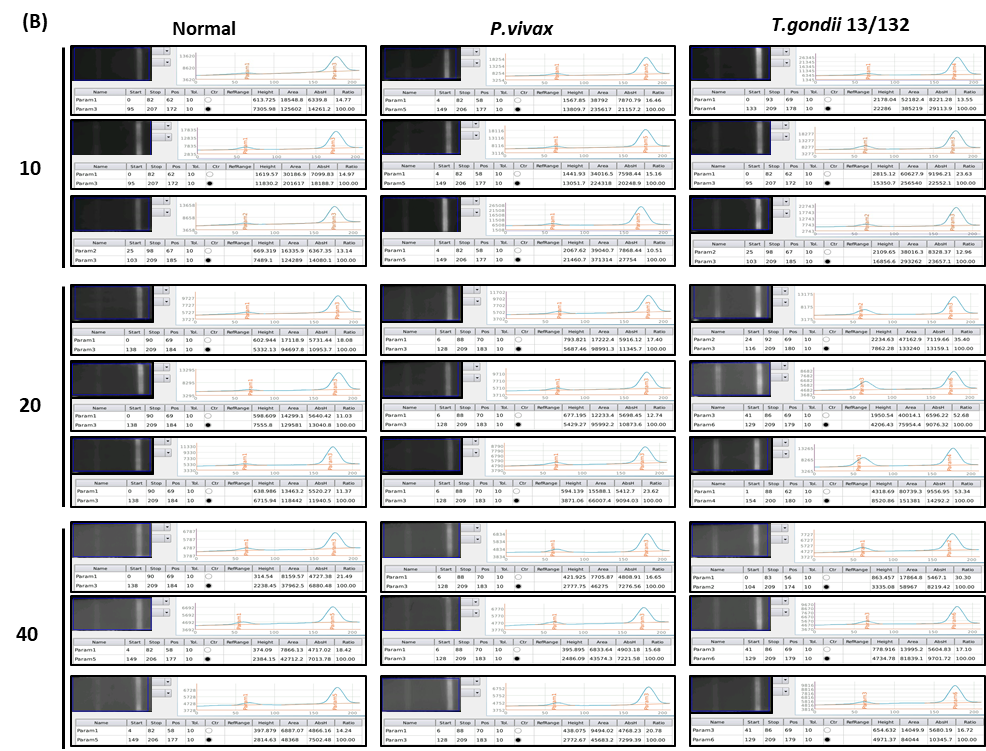


**FIGURE 18-S10. Raw data for Figure 6.**

Immunochromatographic test strip included the test line (TL) coated with 0.3 mg/mL rAg T.g-GAP50 and the control line (CL) coated with 0.05 mg/mL rapid anti-goat IgG (anti-gIgG).

(A) Eu NP-conjugated **anti-mouse** antibodies diluted 40-, 80-, and 160-fold and (B) conjugated **anti-human** antibodies diluted 10-, 20-, and 40-fold were dropped onto the conjugate pad, and the strip was dipped in a mixture of sera for 15 min. The interaction of rAg with Abs present in sera was determined by measuring the fluorescence intensity (365 nm excitation and 610 nm emission). Two microliters of serum were used per reaction.


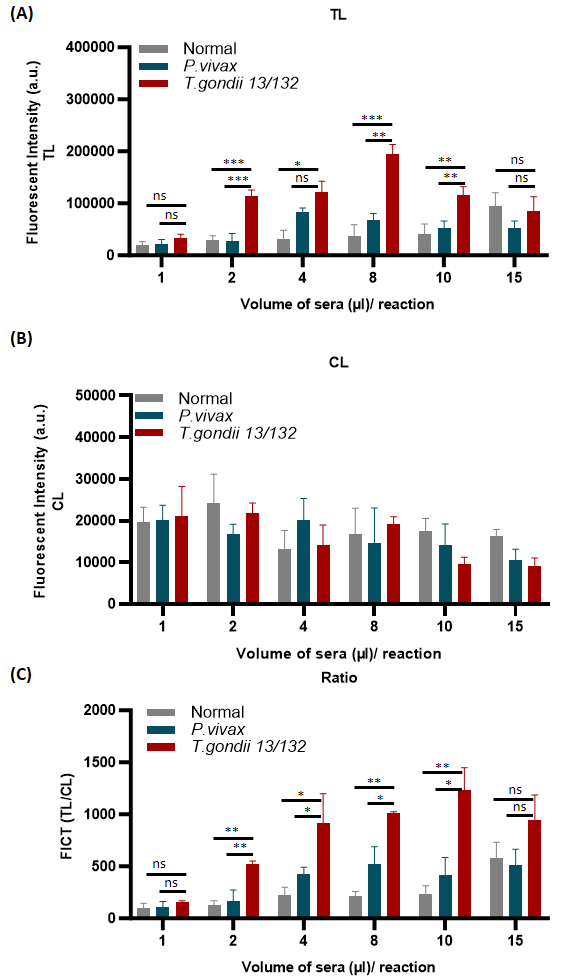


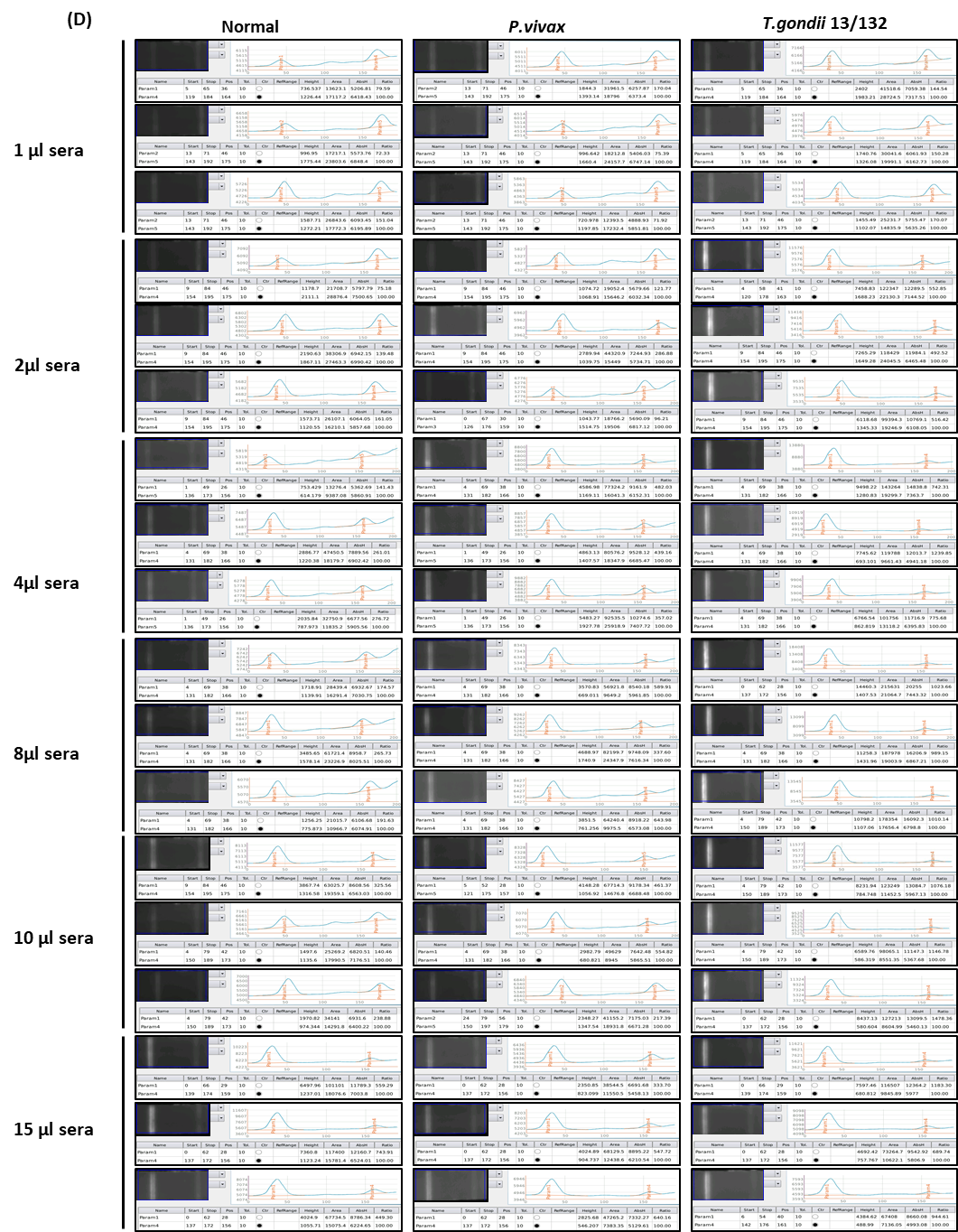


**FIGURE 19-S11. Determination of the optimal volume of sera required per reaction for FICT.**

Various quantities (1, 2, 4, 8, 10, and 15 µL) of sera samples, including normal, *P. vivax*, and standard *T. gondii*-infected human sera (code 13/132) were diluted in distilled water (DW) to yield a total of 75 µL serum sample for each strip reaction. A portable fluorescence detector (excitation at 365 nm and emission at 610 nm) was used to measure the fluorescence signals of the (A) TL, (B) CL, and (C) TL/CL mice. The fluorescent density of TL, CL and the ratio of TL/CL data (*n* =3) was shown as means ± SD.

ns: not statistically significant, **P* < 0.05, ***P* < 0.01, ****P* < 0.001, *****P* < 0.0001.

(D) Raw data from strip tests.


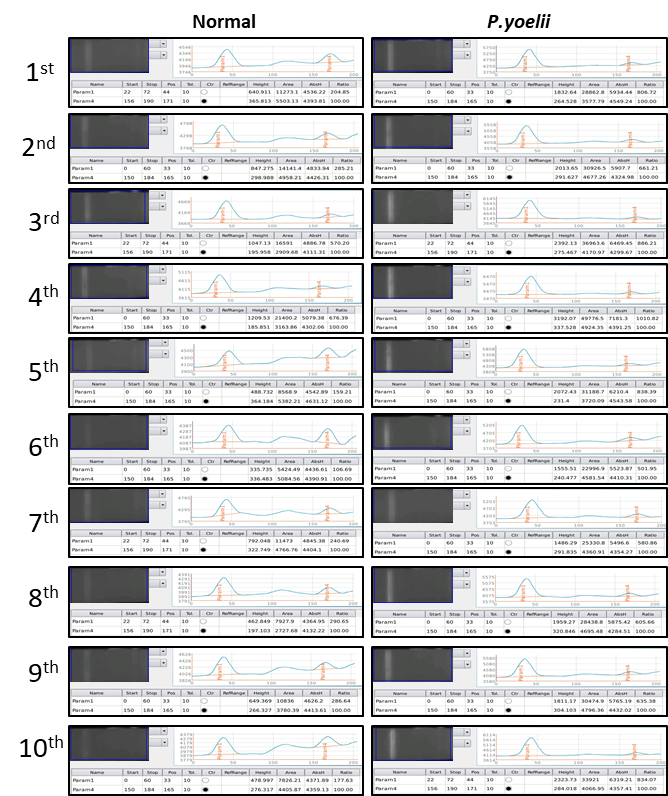


**FIGURE 20-S12. RAW DATA for Figure 7A.** **Determination of the FICT threshold value using the TL/CL ratio.**

Normal and *P. yoelii* (each group, *n* = 10) were evaluated. The cut-off value of the FICT was determined by calculating the mean of the normal sera (*n* = 10) plus three times the standard deviation (SD) using the TL/CL value when applying 2 µL sera per strip reaction. The dotted line indicates that the cut-off value was 867.1.

**
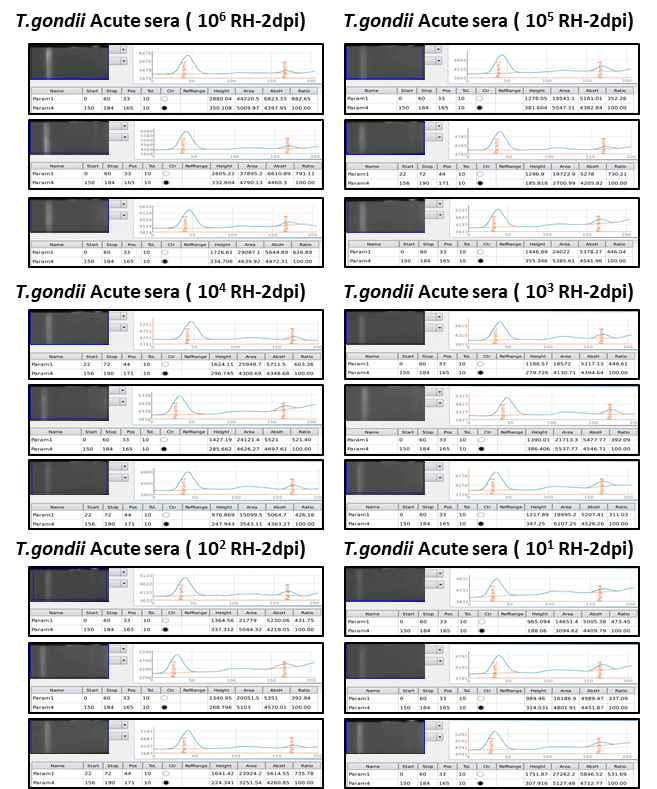
**


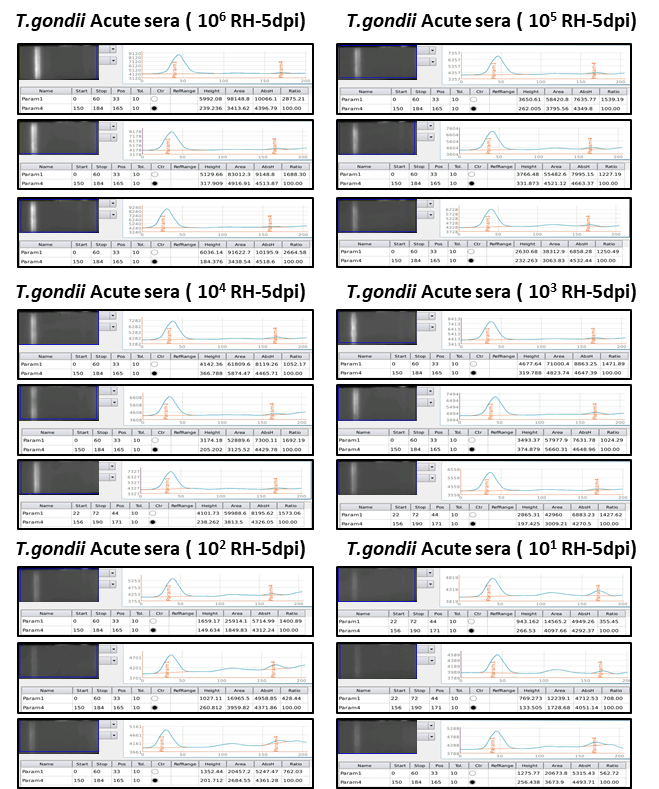


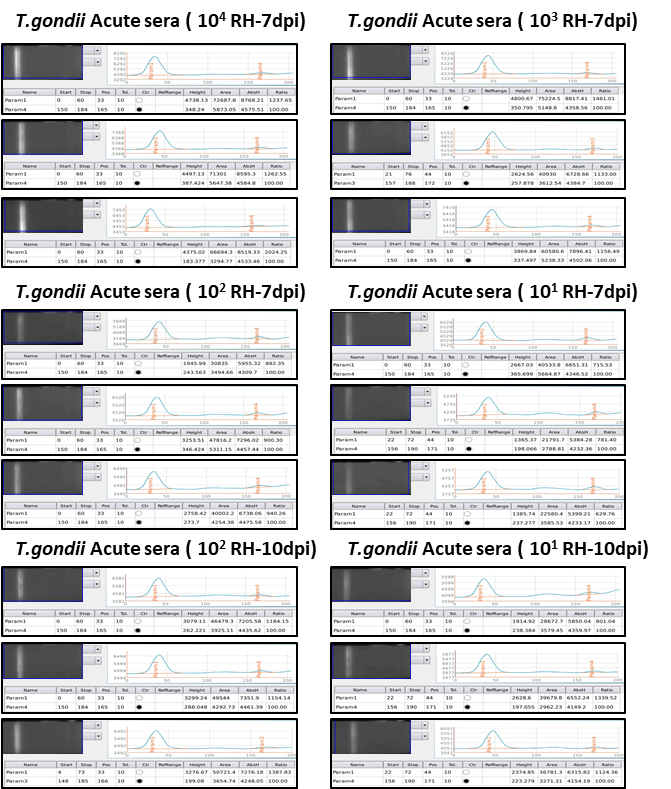


**FIGURE 21- S13. Supplementary raw data for Fig 7.**

Determination of the FICT threshold value using the TL/CL ratio for mouse serum. *T. gondii* (10^1^ – 10^6^)-infected serum of BALB/c mice (each group, *n*=3) at 2, 5, 7, 10 dpi were subjected to the FICT.


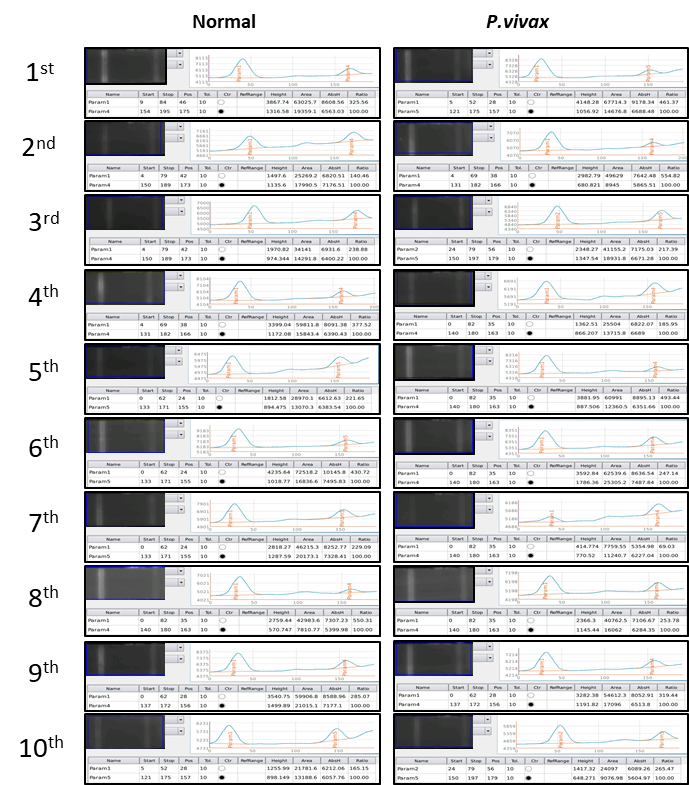


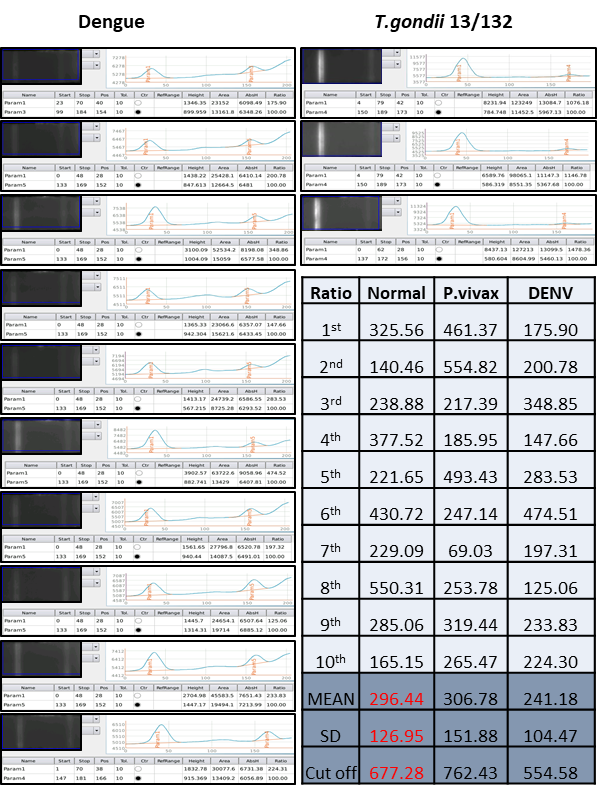


**FIGURE 22-S14. Raw data for Figure 8A. Determination of the FICT threshold value using the TL/CL ratio for human serum.**

Normal and *P. vivax*, and dengue sera (each group, *n* = 10) were evaluated. The cut-off value of the FICT was determined by calculating the mean of the normal sera (*n* = 10) plus three times the standard deviation (SD) using the TL/CL value when applying 10 µL sera per strip reaction. The dotted line indicates that the cut-off value was 677.28 for distinguishing *T. gondii* serum.


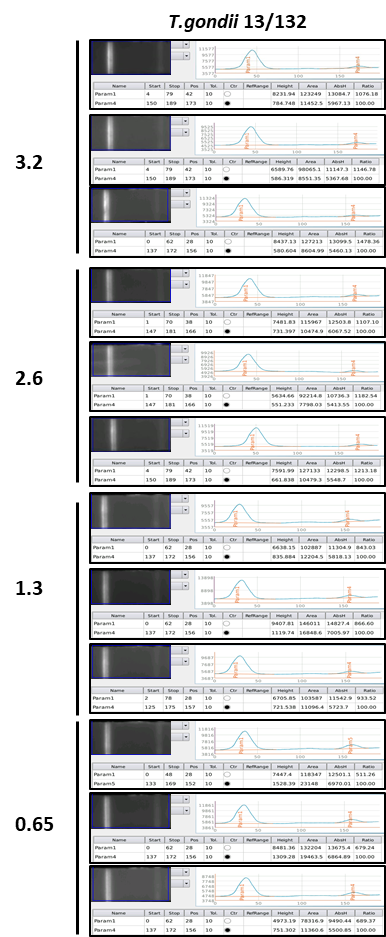


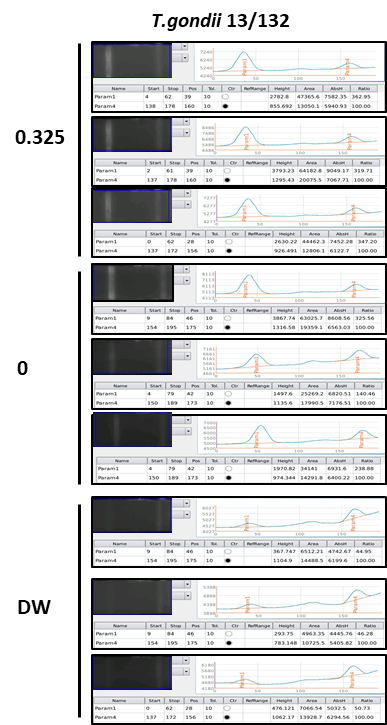


**FIGURE 23-S15. Raw data for Figure 8B. Determination of the FICT LOD.**

(B) *T. gondii 13/132* was prepared by spiking in 10 µL normal sera, which was then subjected to FICT. LOD for the FICT test using a coating of rAg Tg-GAP50 was established at 1.3 IU, equivalent to 4 μL tested.


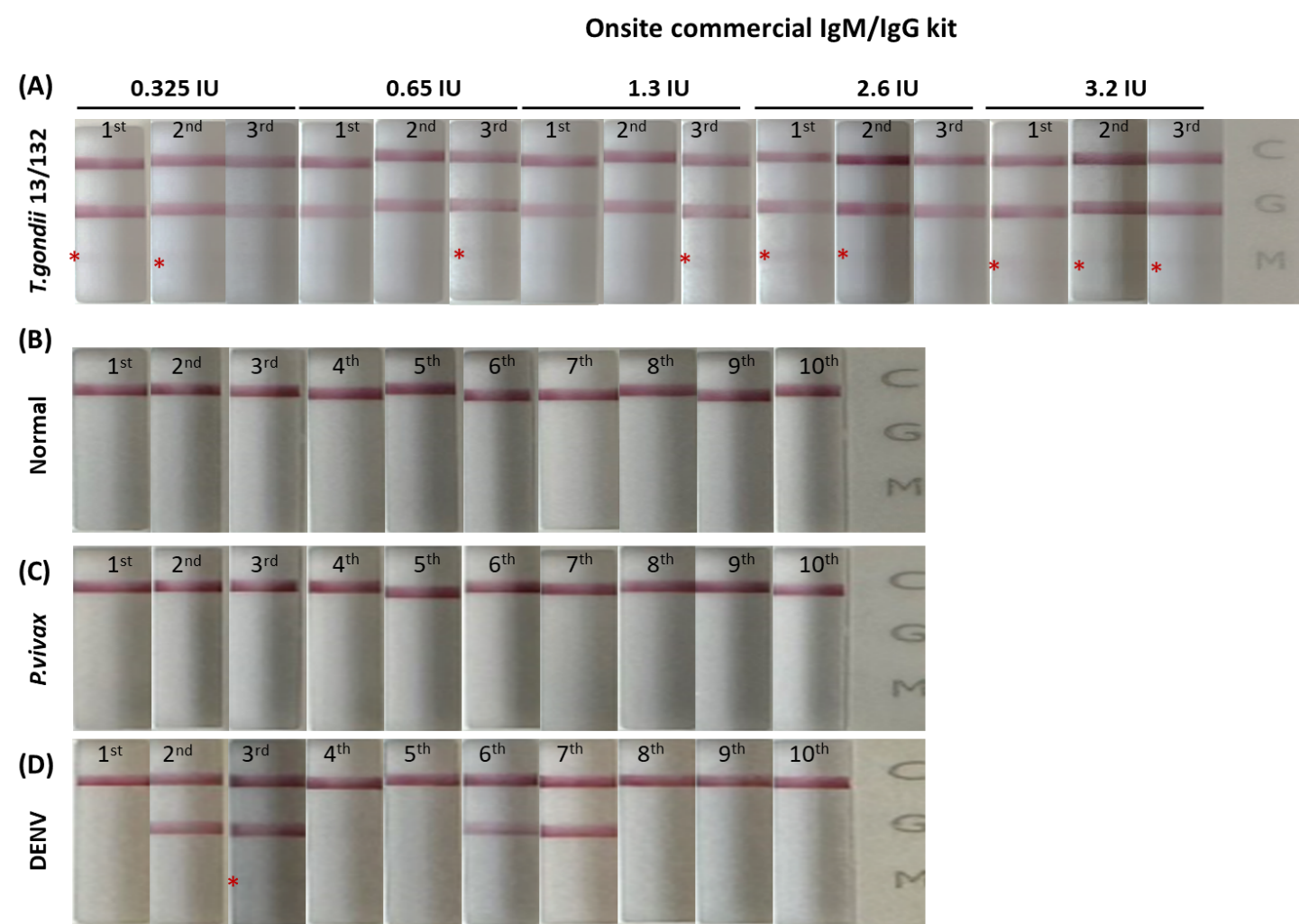


**FIGURE 24. S16. Onsite commercial kit for IgM and IgG serum analysis.**

**A. Onsite commercial kit for IgM and IgG serum analysis using WHO serum**

Following the instructions provided by the Onsite commercial kits, standard T. gondii 13/132 sera were tested in triplicate, using 10 µL each, equivalent to 3.2 IU/strip. To assess the limit of detection (LOD) in parallel with FICT, the sera were similarly prepared by spiking them into 10 µL of normal sera, and then analyzed using both the commercial strip and GAP50-FICT simultaneously. * indicate the IgM line is faintly visible to the naked eye.

**(B-D) Onsite commercial kit for IgM and IgG serum detection.**

Normal sera (B) as well as P.vivax (C) and Dengue (D) patient serum (each group, n=10) had been evaluated by the Onsite commercial kits. Sera were tested in triplicate, using 10 µL each strip. * indicate the IgM line is faintly visible to the naked eye.
